# Structures of asymmetric particles of tick-borne encephalitis virus provide insight into flavivirus assembly and maturation

**DOI:** 10.1126/sciadv.aee4765

**Published:** 2026-07-03

**Authors:** Tibor Füzik, Maria Anastasina, Peter Pajtinka, Ausra Domanska, Lauri I. A. Pulkkinen, Lenka Šmerdová, Lucie Nepovímová, Petra Formanová-Pokorná, Petra Straková, Jiří Nováček, Daniel Růžek, Robert Vácha, Sarah J. Butcher, Pavel Plevka

**Affiliations:** ^1^ Central European Institute of Technology, Masaryk University, Brno, Czech Republic.; ^2^ Faculty of Biological and Environmental Sciences, Department of Molecular and Integrative Biosciences, University of Helsinki, Helsinki, Finland.; ^3^ Helsinki Institute of Life Sciences-Institute of Biotechnology, University of Helsinki, Helsinki, Finland.; ^4^ Laboratory of Emerging Viral Infections, Veterinary Research Institute, Brno, Czech Republic.; ^5^ Department of Experimental Biology, Faculty of Science, Masaryk University, Brno, Czech Republic.; ^6^ Institute of Parasitology, Biology Centre of the Czech Academy of Sciences, Ceske Budejovice, Czech Republic.

## Abstract

Flaviviruses are globally distributed human pathogens. However, the mechanisms underlying flavivirus assembly and maturation remain poorly understood. Here, we show that many particles of tick-borne encephalitis virus (TBEV) are asymmetric and lack subsets of surface heterodimers. Immature particles of TBEV contain incomplete spikes, providing evidence that their coats assemble directly from heterodimers of premembrane (prM) and envelope (E) proteins. Exposure of TBEV particles to acidic pH in the Golgi complex promotes maturation. The spikes and herringbone regions in TBEV maturation intermediates are oriented randomly rather than conforming to a common icosahedral symmetry. Consequently, the mature herringbone lattice forms around a randomly oriented nucleation center, expanding by addition of membrane-envelope heterodimers as the spikes disassemble and prMs are cleaved. The observed incompleteness of the protein coats explains, as an alternative to particle breathing, how flaviviruses can be neutralized by antibodies that bind to regions of E proteins normally inaccessible in the spiky or herringbone structures.

## Teaser

Flavivirus maturation is enabled by asymmetric rearrangement of the virus envelope proteins.

## INTRODUCTION

Viruses from the genus *Orthoflavivirus* of the family *Flaviviridae* are the causative agents of important diseases, including tick-borne encephalitis, dengue, Zika, and yellow fever, and cause major health and economic damage worldwide (*1*). Tick-borne encephalitis virus (TBEV; *Orthoflavivirus encephalitidis*) is endemic in large parts of Europe and Asia (*2*). While most TBEV infections are asymptomatic, some can lead to potentially fatal neurological complications, accounting for at least 10,000 clinical cases and numerous deaths annually (*3*–*5*). TBEV vaccination rates in many affected regions are low, and no specific antiviral treatments are available (*6*).

Flavivirus virions (mature infectious particles) consist of a nucleocapsid, containing the viral single-stranded RNA genome and capsid proteins, which is surrounded by a lipid membrane bearing a coat of transmembrane proteins (*7*–*12*). Flaviviruses assemble as immature particles, which differ from virions in the organization of coat proteins. Immature particles contain heterodimers of premembrane (prM) and envelope (E) proteins, each anchored in the membrane by one peripheral and two transmembrane helices (*13*–*17*). Three such heterodimers form an asymmetric spike protruding from the particle surface. In contrast, flavivirus virions have a smooth surface covered by heterodimers of membrane (M) and E proteins organized in a herringbone pattern (*7*–*9*). The prM-E heterodimers in the icosahedral asymmetric unit of immature particles and M-E heterodimers in the icosahedral asymmetric unit of virions form unique interactions with their neighbors (*7*–*9*, *13*–*15*).

The assembly of flavivirus particles is initiated by the budding of the nucleocapsid into the endoplasmic reticulum (ER) membrane (*18*–*22*). This budding step is linked to the assembly of the prM-E spike coat on the outside of the particle membrane. Host cell membrane remodeling factors enable scission of flavivirus particles from the ER membrane (*23*). Therkelsen *et al.* (*24*) used asymmetric reconstruction to show that a subpopulation of immature particles of the Kunjin strain of West Nile virus has an asymmetrically positioned nucleocapsid and contains a region of poorly resolved envelope protein structure. Immature flavivirus particles are transported via retrograde vesicle transport from the ER to ER-Golgi intermediate compartment (ERGIC), Golgi, and trans-Golgi network (*25*), where the acidic pH promotes the rearrangement of spikes into herringbone patterns and cleavage of prM by the host protease furin (*16*, *26*–*28*). Progeny flavivirus particles are released from cells by exocytosis (*16*, *26*). In addition to mature particles, flavivirus infections produce subpopulations of maturation intermediates that contain separate regions of spiky and herringbone structures (*15*, *26*, *29*–*33*).

Here, we build on previous work analyzing maturation intermediates of dengue virus (*32*, *34*). We present the asymmetric structures of immature particles and virions of TBEV and show that they lack a subset of prM-E or M-E heterodimers. The asymmetric structures of immature particles, maturation intermediates, and virions provide insights into the mechanisms of flavivirus assembly and maturation.

## RESULTS AND DISCUSSION

### Immature particles of TBEV are asymmetric and differ in the content of prM-E

**Fig. 1.**
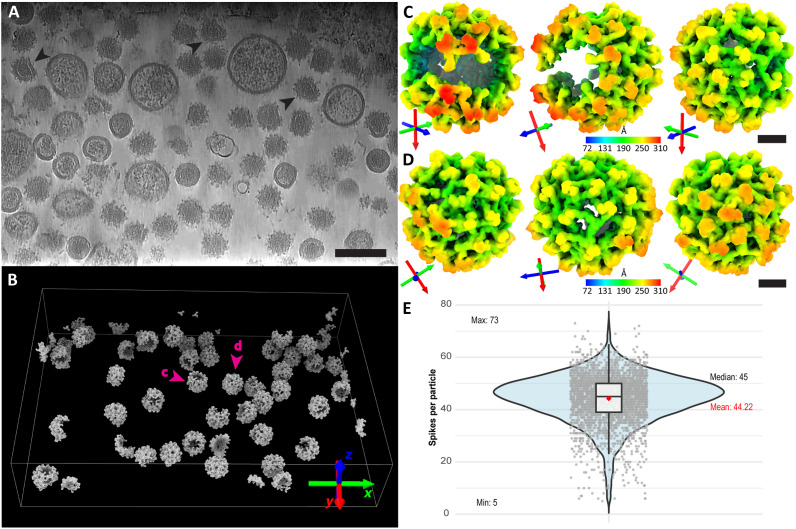
Immature particles of TBEV are asymmetric and miss variable amounts of prM-E heterodimers. (**A**) Projection image of a 1.1-nm-thick section of a cryo-tomogram of immature TBEV particles. Black arrowheads indicate immature particles with exposed membranes. Scale bar, 100 nm. (**B**) Distribution of prM-E spikes in the cryo-tomogram of immature TBEV shown in (A). The positions and orientations of the spikes were determined by template matching, using the cryo-EM density of prM-E spike from single-particle reconstruction low-pass–filtered to 24 Å as a search model. Magenta arrowheads indicate particles shown in detail in (C) and (D). (**C** and **D**) Distribution of prM-E spikes at the surfaces of immature TBEV particles with an incomplete (C) and a complete (D) set of prM-E spikes. Isosurface representations of prM-E spike distributions within particles are rainbow-colored according to distance from particle center. Scale bars, 10 nm. (**E**) Volin plot of prM-E spike content in immature TBEV particles. Individual particles are represented as gray dots. The included boxplot displays median as a bar, mean as a red dot, interquartile range (Q1 to Q3) as a box, and 1.5× interquartile range as whiskers. A total of 2990 particles from 193 tomograms were analyzed.

Electron micrographs of immature TBEV and other flaviviruses show that many of the particles are asymmetric and contain regions of disorganized or missing coat proteins (Fig. 1A) (*13*, *24*). Template matching of prM-E spikes into cryo–electron tomograms of immature TBEV shows that the particles differ in the extent of their surfaces covered by the spikes (Fig. 1, B to D). Ninety-eight percent of immature particles analyzed contained fewer than 60 prM-E spikes, and half of the immature particles had less than 45 spikes (Fig. 1E). Because of the missing spikes, patches of membranes at the particle surface are exposed (Fig. 1, A to C). The orientations of the asymmetric particle features in tomograms were random (Fig. 1B), indicating that the interaction with the air-water interface in cryo–electron microscopy (cryo-EM) samples did not cause the observed asymmetric features.

**Fig. 2.**
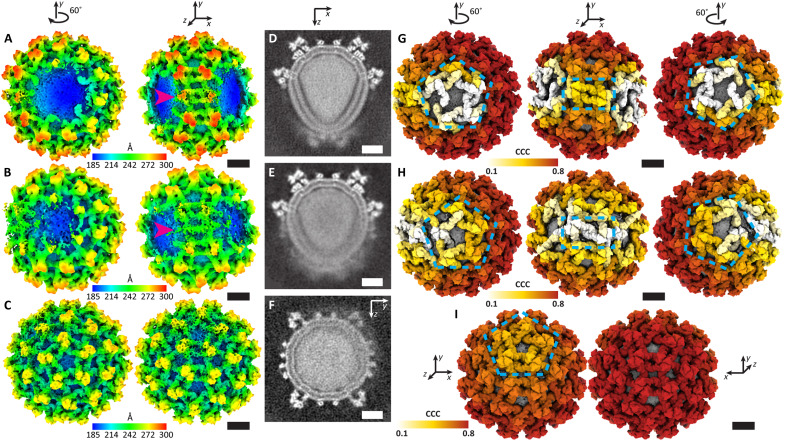
Structures of asymmetric immature TBEV particles. (**A** to **C**) Surface representations of cryo-EM reconstructions of asymmetric immature TBEV particles, radially colored. Particles with two exposed membrane areas (A) and (B), and a particle with a disturbed structure in one region of the coat (C). The particles in (A) and (B) differ in the content of prM-E in the exposed membrane areas. (**D** to **F**) Distribution of cryo-EM densities in central sections of particles. (**G** to **I**) Molecular surface representations of particles with individual E-protein ectodomains colored according to the cross-correlation of their calculated density distribution with local cryo-EM density. Extra dimers disturbing the icosahedral arrangement of prM-E spikes are indicated by a magenta arrowhead in (A) and (B) and blue dashed rectangles in (G) and (H). Blue dashed pentagons highlight exposed membrane areas in (G) and (H) and the region of disturbed prM-E spike structure in (I). CCC indicates cross correlation coefficient. Scale bars, 10 nm.

The classification included in the process of the asymmetric single-particle reconstruction identified immature TBEV particles with one or two patches of exposed membrane (Fig. 2; figs. S1 to S3; and table S1). The particle with two patches of exposed membrane lacks two pentamers of prM-E spikes and contains two additional prM-E spikes inserted between the two membrane patches (Fig. 2, A, B, G, and H). The two extra prM-E spikes interact with each other as if they were a part of a pentamer of spikes (Fig. 2, A and B, and fig. S4). The absence of the two pentamers of prM-E spikes and the insertion of the two additional prM-E spikes affect the particle shape, which resembles a wedge rather than a sphere (Fig. 2, A, B, D, and E). The nucleocapsid follows the shape of the particle membrane (Fig. 2, D and E). The internal structure of the RNA genome is not resolved in the asymmetric reconstructions of immature TBEV (Fig. 2, D to F), indicating that the organizations of the nucleocapsids differ among particles. The shape of the particle deviates from a sphere because the regions of the membrane that are not covered by prM-spikes are flatter than those that are covered. The membrane bending is caused by the perimembrane and transmembrane helices of prM and E, as discussed in detail below. The class of immature TBEV particles that lacks two pentamers of prM-E spikes can be subclassified based on the content of prM-E in the areas of the missing pentamers of spikes (Fig. 2, A, B, G, and H). Icosahedral ordering of complete spikes in these areas is not possible due to the two extra spikes incorporated between the exposed membrane patches, which disrupts the icosahedral symmetry of the particle (Fig. 2, G and H, and fig. S4).

The reconstruction of a particle with one exposed membrane patch has an overall spherical shape (Fig. 2, C, F, and I), and it resembles the asymmetric reconstruction of an immature West Nile virus (WNV) particle with perturbed structure of envelope glycoproteins that was characterized previously by Therkelsen *et al.* (*24*). Nevertheless, the TBEV particle with one exposed membrane patch contains a continuous membrane covering the whole nucleocapsid (Fig. 2F). Particles with incomplete sets of envelope glycoproteins were observed also for alphaviruses that form by budding from the cytoplasmic membrane (*35*).

The budding of flavivirus particles into the lumen of the ER is terminated by membrane scission, a process assisted by host cell factors (*23*). For steric reasons, membrane scission prevents incorporation of a complete icosahedral set of prM-E spikes into the forming particles. The protein-free membrane region of an immature TBEV particle may coincide with the scission site (*24*). Because each particle can contain only one scission scar, we propose that the immature particles with two exposed membrane areas arise when one patch corresponds to the scission site, whereas the second is the product of a coat assembly with limited number of available prM-E heterodimers.

### Coats of immature TBEV particles assemble from prM-E heterodimers

**Fig. 3.**
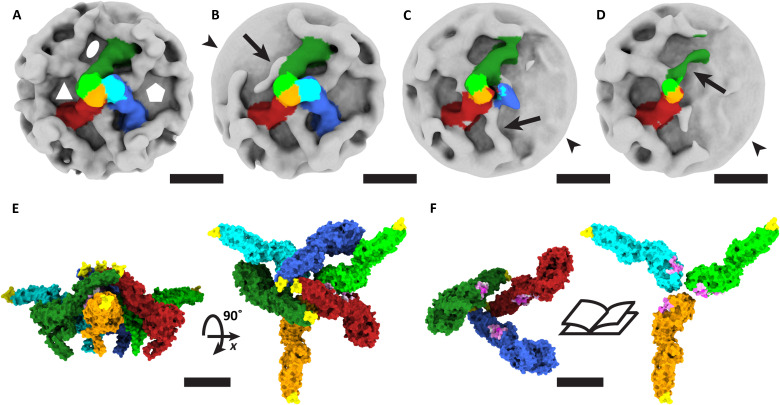
Immature TBEV particles contain incomplete prM-E spikes. (**A** to **D**) Isosurface representation of prM-E spike reconstructions obtained by subtomogram averaging. (A) prM-E spike in a complete icosahedral arrangement of surrounding molecules. Three prM-E heterodimers forming a spike are highlighted in blue, green, and red. E proteins are shown in red, blue, and green and prM in orange, cyan, and light green. The positions of the icosahedral symmetry axes are depicted by a pentagon for a fivefold axis, a triangle for a threefold axis, and an oval for a twofold axis. [(B) to (D)] Incomplete spike structures. Black arrows mark prM-E heterodimers from incomplete spikes. Black arrowheads indicate areas of exposed particle membrane. (**E**) Molecular surface representation of E proteins from a spike and E proteins from neighboring spikes that interact with them. The E proteins forming the spike are shown in red, blue, and green, and the interacting E proteins from neighboring spikes are shown in cyan, orange, and light green. Residues forming the fusion loops are highlighted in yellow. prM proteins are not shown. (**F**) “Open book” representation of buried surface areas of interspike interfaces. The interfaces are colored from white to magenta, with darker colors representing a higher fraction of buried surface areas per residue. Numeric information on the buried surface areas is provided in fig. S9. Scale bars, 5 nm.

Asymmetric reconstructions of immature TBEV particles, determined using single-particle analysis, reveal incomplete prM-E spikes that lack one or two prM-E heterodimers (Fig. 2, G and H, and fig. S3B and S4B). These incomplete spikes occur at the borders of the exposed membrane regions (Fig. 2, A and B, and fig. S4B). In contrast, complete prM-E spikes in asymmetric immature particles remain structurally consistent with those previously described from icosahedral and subparticle reconstructions (figs. S5 and S6, A to C; and table S1) (*13*). Subtomogram averaging combined with classification likewise identified both complete and incomplete prM-E spikes (Fig. 3, A to D; figs. S6, D to F, S7, and S8; and table S1). The prM-E heterodimers that lack interaction partners within a spike are stabilized through contacts with prM-E from neighboring spikes (Fig. 3, B to D), mediated primarily by domains I and III of the E proteins, with buried surface areas of 370 to 420 Å^2^ (Fig. 3, E and F, and fig. S9). The presence of such orphan heterodimers supports a model in which the immature spiky coat assembles directly from individual prM-E heterodimers.

### Membrane helices of prM and E induce membrane bending

In immature TBEV particles with two exposed membrane regions, those regions are flat, whereas regions coated by prM-E spikes display pronounced curvature (Figs. 1A and 2, D and E). These curvature differences support a model in which the transmembrane and peripheral membrane helices of prM and E drive membrane bending. In agreement with previous structural analyses of flaviviral membrane helices (*36*), our molecular dynamics simulations corroborate that peripheral membrane helices of prM/M and E induce positive curvature in the outer leaflet and negative curvature in the inner leaflet of the bilayer (fig. S10). In addition, the transmembrane helices of prM and E promote local membrane thinning by engaging with their charged residues with phosphates in the opposing leaflet (fig. S10). Such membrane bending is required for budding of immature TBEV particles into the ER. The ability of prM-E heterodimers alone to generate sufficient curvature is further supported by the formation of flavivirus-derived virus-like particles lacking capsid proteins and RNA (*37*–*42*).

### Icosahedral symmetries of spiky and herringbone patterns of coat proteins of TBEV maturation intermediates are misaligned

**Fig. 4.**
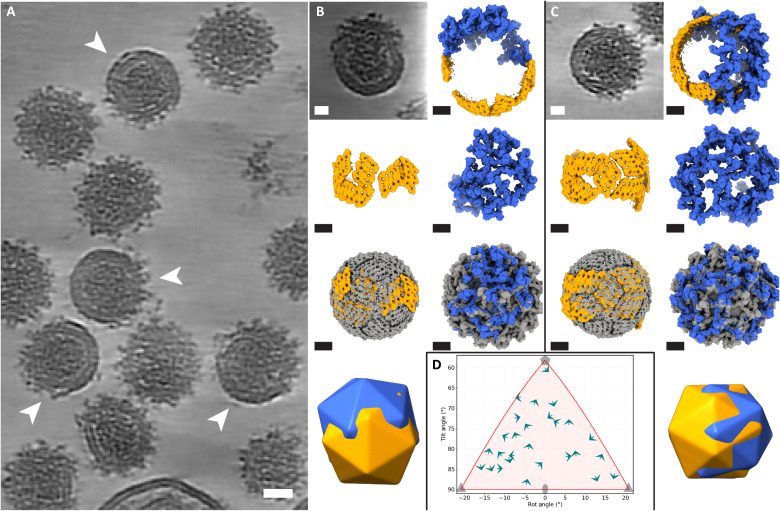
The icosahedral symmetries of spiky and herringbone patterns of TBEV maturation intermediates are oriented arbitrarily relative to each other. (**A**) Projection image of a 1.1-nm-thick section of a cryo-tomogram of TBEV particles. White arrowheads indicate maturation intermediates containing regions of spike and herringbone structures. Scale bar, 20 nm. Panels 1 and 3 of fig. S11 show identical particles to those displayed in this overview. (**B** and **C**) Template-matching analysis of TBEV maturation intermediates. Top left: Projection images of 1.1-nm-thick sections of cryo-tomograms of TBEV maturation intermediates. Top-right: Three-dimensional representations of positions of herringbone rafts (orange) and prM-E spikes (blue) identified using template matching. The fitting algorithm identified the placement of the herringbone patterns with confidence only when their orientations were approximately perpendicular to the *xy* plane of the tomogram. Second row: Rotated views showing the arrangements of herringbone patterns and spikes. Third row: Overlays of the individually placed herringbone patterns and spikes with aligned icosahedral mature and immature TBEV structures (gray). Scale bars, 10 nm [(B) and (C)]. Bottom row: Blue and orange icosahedra show the relative orientations of the icosahedral symmetries of the partial immature (blue) and mature (orange) patterns. Analysis of additional TBEV maturation intermediates is shown in fig. S11. Scale bars, 10 nm. (**D**) Plot of relative orientations of icosahedral symmetries of immature and mature domains in TBEV maturation intermediates. The immature symmetry of all the particles was brought to a standard icosahedral orientation, as indicated by the red triangle outlining the icosahedral asymmetric unit. Position of the fivefold, threefold, and twofold symmetry axes are indicated by pentagon, triangle, and oval, respectively. Orientations of mature domains, relative to the immature domains in the standard orientation, are indicated by positions (rot and tilt) and rotations (psi) of arrows.

TBEV particles released from infected cells include maturation intermediates containing regions of immature spiky and mature herringbone structures (Fig. 4A). Although these chimeric particles may represent dead-end products of maturation, similar intermediates have been reported for multiple other flaviviruses (*26*, *29*, *32*, *43*, *44*). Cryo–electron tomography (cryo-ET) combined with template matching of individual prM-E spikes and M-E herringbone rafts shows that these intermediates differ in the relative proportions of membrane surfaces occupied by the spiky and herringbone structures (Fig. 4, B and C, and fig. S11). We did not detect any consistent relationship between the orientations of the immature and mature domains (*P* = 0.76; Fig. 4D and fig. S11). The random spatial relationship between these domains indicates that the reorganization of spikes into the herringbone lattice does not proceed within the icosahedral symmetry framework of the immature structure. Instead, spike trimers disassemble while the heterodimers remain membrane-anchored, allowing the herringbone lattice to nucleate and assemble in an arbitrary orientation relative to the original symmetry of the immature structure. An alternative possibility is that partially formed mature domains undergo rearrangement after initial assembly; however, this would require two mechanistically distinct transitions—one preserving the immature icosahedral framework and another disregarding it, which we consider unlikely.

**Fig. 5.**
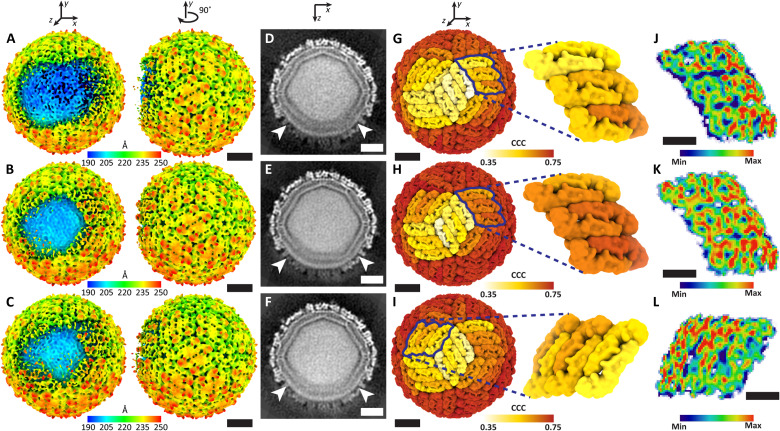
Structures of TBEV virions with incomplete sets of coat proteins. (**A** to **C**) Isosurface representations of cryo-EM reconstructions of TBEV virions missing subsets of M-E heterodimers. The particles are colored radially based on the distance from the particle center. The areas of exposed membrane are shown in blue. (**D** to **F**) Distributions of cryo-EM densities in central sections of the reconstructions of asymmetric TBEV virions. White arrowheads indicate the borders of the exposed membrane areas. (**G** to **I**) Molecular surface representations of the virions with individual E-protein ectodomains colored according to their cross-correlation with local cryo-EM density distribution. Details of selected herringbone rafts are shown at a higher magnification. (**J** to **L**) Cryo-EM densities 222 nm from particle center, corresponding to the herringbone rafts shown in magnified detail in (G) to (I), respectively. The whole particles are shown in fig. S17. Scale bars, 10 nm [(A) to (I)]; 5 nm [(J) to (L)].

Single-particle analysis and classification show that many TBEV virions deviate from icosahedral symmetry and contain exposed membrane patches (Fig. 5; figs. S12 to S15; and table S1). These uncovered membrane regions are typically ellipsoidal and vary in size, corresponding to 18 to 36 missing M-E heterodimers (Fig. 5, A to C, and figs. S12 and S13). In addition, there is a small subset of particles with two domains of smooth arrangement of E proteins (fig. S16). As in immature particles, exposed membrane regions are flatter than those covered by the herringbone pattern of M-E heterodimers (Fig. 5, D to F). Cryo-EM density values differ among neighboring M-E heterodimers at the borders of these regions (Fig. 5, G to L, and figs. S13 and S17), suggesting that there are multiple structural variants of incomplete virions, distinct in heterodimer content and arrangement. Because classification could not separate these variants into homogeneous groups, the resulting reconstructions contain residual heterogeneity that manifests as variation in the density of individual M-E heterodimers (Fig. 5, G to L).

Cross-correlation with a template, occupancy analysis, and comparison of map intensity levels indicate that the presence of neighboring E proteins in herringbone rafts is independent of each other (Fig. 5, G to L, and figs. S13 and S17). This provides indirect evidence that the herringbone patterns of TBEV are assembled by the addition of individual M-E heterodimers. Furthermore, based on single-particle analysis and classification, 68% of immature TBEV particles contain two patches of exposed membrane; however, this was not the case in the asymmetric virion reconstructions (fig. S12). The difference in the shapes of the exposed membrane areas between immature particles and virions suggests that maturation involves long-distance lateral movements of heterodimers within the particle membrane.

Reorganization of flavivirus coat proteins from a spiky to a herringbone arrangement occurs in the lumen of the ERGIC, Golgi, and trans-Golgi network, where the increasingly acidic pH (*25*) enables the cleavage of prM by the host protease furin (*16*, *26*–*28*). However, the furin cleavage site in prM is inaccessible to furin both in the prM-E spikes (*13*–*17*) and in the herringbone structure (*7*, *17*, *45*). Our results provide evidence that prM-E spikes disassemble and that the herringbone pattern assembles gradually from a nucleation center by adding M-E heterodimers to its edges. Previously, it was proposed that prM furin cleavage requires disassembly of the immature particles (*16*, *46*). We speculate that the furin cleavage occurs during the interval between prM-E spike disassembly and M-E heterodimer incorporation into the herringbone pattern, during which the surrounding structures do not cover the cleavage site.

### Flavivirus assembly and maturation mechanisms

**Fig. 6.**
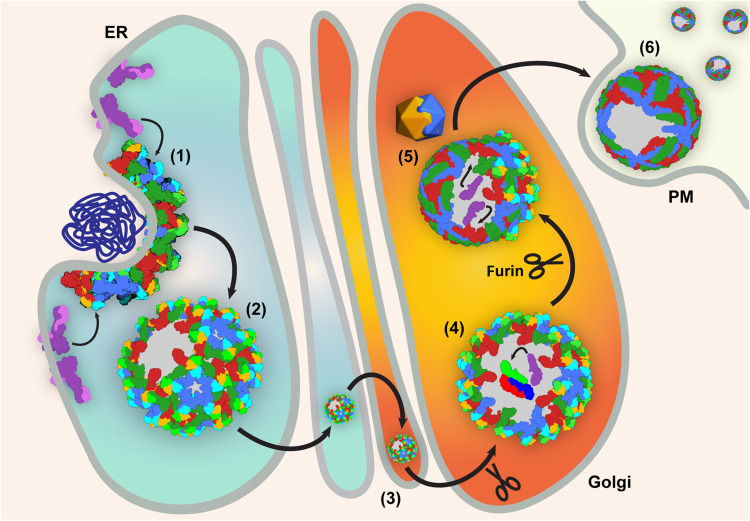
Scheme of TBEV assembly and maturation. (1) Immature TBEV particles form by budding into the lumen of the ER. The icosahedral arrangement of prM-E spikes (red, green, and blue) is assembled from prM-E heterodimers (magenta). E proteins are shown in darker colors, and prM in lighter colors. (2) The budding process is completed by particle scission, which results in a particle with an area of exposed membrane that is not covered by prM-E spikes. (3) Immature particles are transported to the Golgi complex and encounter acidic pH, which promotes their maturation. (4) prM-E spikes disassemble, which may enable cleavage of prM by the furin protease. The herringbone pattern assembles from an arbitrarily oriented nucleation center from M-E heterodimers. (5) TBEV maturation intermediates contain domains covered by icosahedral arrangements of spikes and herringbone patterns, which are arbitrarily oriented relative to each other rather than being aligned to one icosahedral symmetry. (6) Mature virions, containing regions of exposed membranes, are released from cells by exocytosis.

Cryo-EM and cryo-ET analyses of TBEV immature particles and virions reveal that they are asymmetric and that they lack subsets of coat proteins, resulting in exposed areas of the particle membrane. The structural characterization of these asymmetric particles provides indirect evidence regarding the mechanisms of flavivirus assembly and maturation. Particle budding is enabled by the localized accumulation of prM and E in the ER membrane because the membrane helices of the proteins induce membrane bending (Fig. 6) (*36*). Some prM-E heterodimers in immature particles are not incorporated into spikes, which provides evidence that the immature arrangement of spikes assembles directly from prM-E heterodimers (Fig. 6). Scission of flavivirus particles from the ER membrane is mediated by cellular factors (*23*). We speculate that, due to topological constraints, membrane scission produces immature particles with incomplete sets of coat proteins.

The structures of prM-E spike and M-E herringbone pattern of flaviviruses are distinct, and the mechanism of the reorganization of one to the other has been the subject of extensive speculation (*13*, *17*, *32*, *34*, *45*, *47*). Most previously proposed maturation mechanisms assumed the preservation of the icosahedral symmetry framework from the immature spiky structure to the herringbone arrangement (*17*, *45*, *47*). However, our results confirm that the icosahedral symmetries of spiky and herringbone patterns in TBEV maturation intermediates are arbitrarily oriented relative to each other (Figs. 4, B to D, and 6) (*32**,* *34*). Therefore, the maturation-related reorganization of spikes into the herringbone pattern does not occur within a single framework of icosahedral symmetry. Instead, the herringbone pattern assembles from a nucleation center that is oriented randomly relative to the symmetry of the immature structure (Fig. 6). The variation in the occupancy of neighboring M-E heterodimers at the borders of the exposed membrane patches of TBEV virions indicates that the herringbone patterns assemble by the addition of individual heterodimers (Fig. 6). The shapes of the exposed membrane areas in immature and mature TBEV particles differ, indicating that the maturation-related reorganization involves long-distance movements of the coat proteins in the particle membrane (Fig. 6). Therefore, we propose that there is no single maturation path that determines which heterodimer from a spike will occupy which position in a mature herringbone pattern. Instead, heterodimers diffuse in the particle membrane and attach to the edges of the forming mature structure (Fig. 6). The release of prM-E heterodimers from spikes during the reorganization and/or protein dynamics on the edge of the membrane patch may expose the prM target site to furin cleavage. The exposed membrane areas in TBEV particles may facilitate the reorganization from a spiky to a herringbone structure by providing space for lateral heterodimer movement in the particle membrane.

Most structurally characterized flaviviruses form virions built from a continuous herringbone arrangement of M-E heterodimers (*7*–*11*). The absence of a fraction of M-E heterodimers from the virion surface, as revealed in asymmetric reconstructions of TBEV virions, is unlikely to compromise infectivity because the particles retain sufficiently large regions of the herringbone pattern to support efficient receptor binding (*48*). The exposed membrane patches may promote fusion during entry by facilitating the formation of close contacts between the viral and endosomal membranes. The structures of incomplete immature particles and virions of TBEV provide a mechanistic explanation for how flaviviruses can be neutralized by antibodies targeting epitopes that are inaccessible on fully formed spiky and herringbone lattices (*49*). Antibody binding at intersubunit interfaces could also account for cross-flavivirus reactivity, as residues involved in intersubunit interactions are generally more conserved than those displayed on the particle surface. Consequently, incomplete particles may contribute to the occurrence of antibody-dependent enhancement, which is often mediated by weakly binding cross-reactive antibodies (*50*). For dengue virus, epitopes associated with antibody-dependent enhancement occur in quaternary epitopes of the virion, the fusion loop, and in prM (*51*). Incomplete flavivirus particles expose at their surface epitopes that would be buried in particles with complete icosahedral shells, a structural state that functionally parallels the previously described particle breathing phenomenon (*52*).

## MATERIALS AND METHODS

### Cells and viruses

Human neuroblastoma SK-N-SH cells [American Type Culture Collection (ATCC) HTB-11] were maintained in Dulbecco’s modified Eagle’s medium with glucose (1 g/liter) (DMEM; Sigma-Aldrich) supplemented with 10% fetal bovine serum (FBS; Gibco), penicillin (0.5 mg/ml) and streptomycin (500 U/ml) (penstrep; Lonza Bioscience), 2 mM GlutaMAX (Gibco), and nonessential amino acids (Gibco). Baby hamster kidney cells (BHK-21, ATCC CCL-10) were maintained in DMEM (Sigma-Aldrich) supplemented with 10% FBS (Sigma-Alrich). All cells were maintained at +37°C in 5% CO_2_ atmosphere. TBEV strain Neudoerfl (European subtype; GenBank U27495.1) was passaged several times in the brains of suckling mice, in UKF-NB4 and BHK-21 cells before its use in the present study. The virus was provided by F. X. Heinz, Medical University of Vienna. Virus titers were estimated by plaque assay, as described previously (*53*).

### Production and purification of immature TBEV particles

For production of immature particles, BHK-21 cells were grown to 85% confluency and infected by TBEV Neudoerfl at a multiplicity of infection (MOI) of 1. The cells (30 flasks with 300-cm^2^ surface area) were incubated in medium (DMEM, 5% FBS, and 25 mM Hepes, pH 7.4) for 26 hours. The medium was replaced by medium containing NH_4_Cl (DMEM, 2% FBS, and 20 mM NH_4_Cl), and the cells were incubated for 24 hours at 37°C, 5% CO_2_. After incubation, the collected medium was clarified by centrifugation (5700*g*, 4°C, 20 min). Polyethylene glycol, molecular weight 8000 (PEG-8000) dissolved in TNE buffer (40% PEG, 20 mM tris, 120 mM NaCl, and 1 mM EDTA, pH 8.5) was added to the final concentration of 8% (w/v) PEG to the clarified supernatant. The particles were fixed by addition of 0.05% formaldehyde (v/v, final concentration) and precipitated overnight (O/N) in an orbital shaker at 130 rpm, 4°C. The precipitate was pelleted at 15,000*g*, for 60 min at 4°C. The pellet was resuspended in 10 ml of TNE buffer containing 8% PEG-8000 (w/v) and pelleted by centrifugation at 15,000*g* for 60 min at 4°C. The pellet was resuspended in 3 ml of TNE buffer, ribonuclease (RNAse) A was added (10 μg/ml, final concentration), and the solution was incubated for 15 min at 15°C. The suspension was centrifuged at 15,000*g*, 10 min, 4°C, and the supernatant was loaded on 10 to 35% (w/v) potassium tartrate step gradient and centrifuged at 175,600*g*, 2 hours, 4°C. The light-scattering band was collected. The sample was buffer-exchanged into TNE buffer by serial dilution and concentration using Amicon Ultra centrifugal filters (Merck).

### Production and purification of mature TBEV particles

Purified TBEV virions were prepared using a modified protocol described previously (*9*). Human neuroblastoma cells UKF-NB4 were grown to 100% confluence in Iscove’s modified Dulbecco’s medium (IMDM) supplemented with 10% FBS at 37°C in the presence of 5% CO_2_ in 30 flasks, each with a bottom surface area of 300 cm^2^. The cells were infected with the TBEV strain Neudoerfl at an MOI of 0.5. After 5 hours of incubation at 37°C, the medium was replaced with fresh medium without FBS. The culture media were harvested 35 hours postinfection and clarified by centrifugation at 5700*g* for 10 min at 4°C. The particles were then fixed by addition of 0.05% formaldehyde (v/v, final concentration), and the supernatant was precipitated by adding PEG-8000 in TNE buffer to a final concentration of 8% (w/v) and incubating overnight at 4°C with mild shaking. After that, the virus was pelleted by centrifugation at 10,500*g* for 50 min at 4°C. The resulting pellet was resuspended in 2 ml of TNE buffer. The solution was clarified by centrifugation at 1500*g* for 5 min at 4°C. RNAse A was added to the supernatant to a final concentration of 10 μg ml^−1^ and incubated for 15 min at 10°C. The solution was loaded onto a 10 to 35% (w/v) potassium tartrate step gradient in TNE buffer and centrifuged at 175,600*g*, 2 hours, 4°C. An opalescent band containing the virus was harvested using a syringe with a needle. The sample was buffer-exchanged into TNE buffer by serial dilution and concentration using Amicon Ultra centrifugal filters (Merck).

### Cryo-EM sample preparation

The samples of immature or mature TBEV particles were vitrified in liquid ethane on holey carbon-coated copper grids (Quantifoil 2/1, mesh 300, Quantifoil Micro Tools GmbH) using a Vitrobot Mark IV (Thermo Fisher Scientific). Samples were stored under liquid nitrogen until use.

### Cryo-EM single-particle analysis data collection

The single-particle datasets of immature and mature TBEV particles were collected on a Titan Krios microscope (Thermo Fisher Scientific) operating at 300 kV aligned for fringe-free imaging and equipped with a K3 direct electron detector behind an energy filter (BioQuantum K3, Ametek) with a 10 e^−^V slit inserted. The micrographs were collected in counting mode at a nominal magnification of 105,000× resulting in a 0.8336-Å pixel size on the detector. The nominal defocus range applied during the acquisition was −3 to −1 μm, and the total dose during the 2-s acquisition was ~40 e^−^/Å^2^. The dose fractionated acquisitions were saved as 40 fraction movies. SerialEM software (*54*) was used for the data acquisition using the beam-tilt compensation upon image shift. In total, 11,246 movies were collected of immature particles and 13,122 movies of mature particles. Data were collected at the cryo-EM and tomography core facility CEITEC MU (Brno, Czech Republic). Refer to table S1 for the full data collection information.

### Cryo-ET data acquisition

The tilt series were acquired on a Titan Krios microscope (Thermo Fisher Scientific) operating at 300 kV, equipped with a K3 direct electron detector (Ametek) behind a BioQuantum energy filter operating in zero-loss mode with slit width of 10 e^−^V. The detector was operating in counting mode using the correlated double sampling feature. The microscope was aligned for fringe-free imaging with no objective aperture inserted. The tilt series were collected at magnification of 64,000× resulting in a pixel size of 1.346 Å on the detector. For tilt-series acquisition, PACEtomo (*55*) Python script integrated into SerialEM (*54*) was used. A regular pattern of 5 × 5 acquisition areas per set of tilt-series acquisition was set up. Dose symmetric acquisition scheme altering two positive and two negative angle values of stage tilt, with a range of ±60° and tilt step of 3°, was used. Every tilt image was acquired as a 1-s movie fractionated into four fractions. The exposure per tilt was 3.6 e^−^/Å^2^, resulting in a total dose of 147.6 e^−^/Å^2^ on the sample per complete set of tilt series. In total, 193 tilt series were collected. Data were collected at the cryo-EM and tomography core facility CEITEC MU (Brno, Czech Republic).

### Single-particle analysis of immature TBEV spikes

The single-particle analysis of TBEV spikes was performed as described previously (*13*). The collected dose-fractionated movies were aligned using MotionCor2 (*56*), and the sum of the dose-weighted fractions were saved as individual micrographs. Contrast transfer function (CTF) estimation was performed using Gctf v1.06 (*57*). The initial set of particles was hand-picked on 10× binned micrographs, and a crYOLO (*58*) neural network was trained on this subset. Automatic particle picking was performed on 10× binned micrographs using crYOLO, and the resulting coordinates were corrected by the binning factor to match the particle positions on the unbinned micrographs. For initial two-dimensional (2D) classification, the particles were extracted and down-sampled from 960 pixel (px) box size to 128 px (pixel size: 6.25 Å). Several rounds of 2D classification were performed in Relion 4.0.0 (*59*) to exclude false-positive particles picked by crYOLO. The resulting 36,236 particles were re-extracted and down-sampled to a box size of 512 px (pixel size: 1.56 Å). Refinement using a low-pass–filtered (40 Å) initial model from a previous refinement of TBEV Hypr was done in Relion v 3.1.2 (*60*) with icosahedral symmetry applied, using only a spherical mask of diameter 650 Å. No further 3D classification or masked refinement improved the map resolution or quality. The final map was masked by a threshold mask and B-factor sharpened in the Relion 3.1.2 postprocessing procedure. The final resolution was estimated using the Fourier shell correlation at threshold 0.143 (FSC_0.143_) criterion as 7.15 Å. To improve the resolution of the spike trimers, single spike trimers were extracted in a box size of 300 px from the original micrographs by a modified version of the localized reconstruction procedure (*61*). In total, 1,639,578 subparticles were extracted and subjected to initial 3D refinement using local searches around the already known orientations. A soft segment mask that included a single spike trimer together with the membrane was applied in the refinement step. After refinement, three rounds of 3D classification were performed, dividing the particles into 40 classes. The orientation search was omitted during the classification, and the orientations from the previous refinement step were used. The selected classes included 552,993 particles, which were subjected to 3D refinement. This was followed by anisotropic magnification estimation, third and fourth order aberration estimation, and defocus refinement per particle in Relion 3.1.2. To analyze the interspike contacts between the neighboring spikes on the surface of the immature TBEV particle, a soft-edged segment mask was prepared that included the E proteins underneath the spike trimer from the neighboring asymmetric unit. Only domains I and III were included from the neighboring E proteins. Using this mask and the newly introduced Blush regularization in Relion 5.0 (*62*), 3D refinement was performed followed by CTF refinement and Bayesian polishing of the particles. The final Blush regularization–enabled 3D refinement included 552,993 particles.

Last, the volumes were reconstructed using relion_reconstruct applying an Ewald sphere correction, to avoid the Wiener filter applied in the last iteration step of 3D refinement when Blush regularization is enabled. The final map was masked, and B factor was sharpened. The final resolution was estimated using the FSC_0.143_ criterion as 3.53 Å (fig. S6, A to C).

### Single-particle analysis of asymmetric immature TBEV particles

Up to the 2D classification step, the initial steps for data processing were the same as for the single-particle analysis of the immature TBEV spikes. Particles resulting from the 2D classification (36,236) were used for preparation of an asymmetric initial model using the stochastic gradient descent method implemented in Relion 3.1.3 (*60*). The following 3D refinement produced an asymmetric particle. Three-dimensional classification into 10 classes was used to separate the particles. The resulting classes contained particles with variable amounts of spikes missing on the particle surface. Representative classes were merged into two groups of particles representing two different occupancies with 11,571 and 13,214 particles. These groups were 3D refined, and the final maps were postprocessed, leading to final resolutions of 9.41 and 10.25 Å (FSC_0.143_), respectively.

To identify the particles with the highest complement of spikes after the initial 2D classification, two rounds of 3D classification were used to select the particles with a single exposed membrane patch. Final refinement of 4499 particles followed by postprocessing produced a 10.96-Å resolution map (FSC_0.143_) (fig. S3).

### Single-particle analysis of asymmetric TBEV virions

The collected dose-fractionated movies were aligned using motion correction implemented in Relion 3.1 globally and locally in 5 by 5 patches, and the sums of the dose-weighted fractions were saved as individual micrographs. CTF estimation was performed using CtfFind4. Particles were picked on 10× binned micrographs with crYOLO using a model pretrained on 50 micrographs of manually picked particles. For initial 2D classification, the particles were extracted and down-sampled to pixel size 2.55 Å with a box size of 256 px. This classification served to exclude bad particles picked by crYOLO, and the good particles were further 2D-classified into classes representing particles with complete and incomplete protein shells. Both selections were further 2D-classified to remove bad particles. The set of particles with complete protein shells was further subjected to 3D classification to sort out the incomplete particles that were not detectable by 2D classification. These incomplete particles were merged with the incomplete particles from the 2D classifications and subjected to 3D refinement with C1 symmetry applied. A stochastic gradient descent–generated asymmetric map from the incomplete 2D-classified particles was used as an initial model. The final masked and postprocessed map reached 5.1-Å resolution (FSC_0.143_). Heterogeneity analysis of the final particles was done using cryoDRGN 2.3.0 (*63*). First, a neural network was trained on 128-px down-sampled particles over 75 epochs to detect “junk” particles. After removal of bad particles, a neural network was trained on 256-px particles over 150 epochs. The 8D latent space of particle descriptors was clustered into 10 clusters using the Gaussian mixture model (GMM) clustering method implemented in cryoDRGN (*63*). To avoid the possibility of hallucinations introduced by the neural network during volume reconstruction by cryoDRGN, the clusters of particles were exported into STAR format and reconstructed using the relion_reconstruct program. The orientational distribution of the particles in the clusters were plotted and the uniformity of the sampling evaluated using the sampling compensation factor (SCF*) (*64*).

### Model building and molecular interface analysis

The previously built model of immature TBEV prM_3_E_3_ [Protein Data Bank (PDB) ID: 8PUV] (*13*) was rigid body–fitted into the postprocessed electrostatic-potential map together with the domains I and III of the E proteins from the neighboring asymmetric unit located underneath the spike. The model was iteratively refined by manual building in Coot (*65*) and refinements in Isolde (*66*) and Phenix (*67*), while the model geometry was monitored with the MolProbity tool (*68*). The final refinement was done using phenix_realspace_refine from Phenix v1.20. PDBePISA (*69*) was used to calculate the buried surface areas of molecular interfaces. Visualization of the model and the interaction interfaces was done in UCSF ChimeraX (*70*).

### Model-to-map correlation analysis of asymmetric reconstructions of TBEV particles

To evaluate the completeness of the protein coats of mature and immature TEBV particles, we used correlation analysis using the molecular models of the E proteins or prM-E heterodimers in conformations present in the mature or immature particles. For the herringbone structures, E-protein ectodomains from one icosahedral asymmetric unit were rigid body–fitted into the asymmetric reconstructions. Where the map quality was not sufficient for reliable fitting, the surrounding subunits served as a prior for the correct orientation, which was refined by rigid-body fitting. The asymmetric unit was split into individual E proteins, and the correlation of every E protein versus the map was calculated. For the immature particles, the fitting was similar, but we used the prM-E spikes for rigid-body fitting into the asymmetric maps. All the correlation calculations were performed in UCSF ChimeraX (*70*) using custom-made scripts. The molecular surfaces of the models were colored according to the resulting correlation coefficient and displayed using UCSF ChimeraX (*70*).

### Occupancy analysis of asymmetric reconstructions of mature TBEV particles

Occupancy analysis of the asymmetric mature TBEV particle maps was done using the program Occupy (*71*), using a kernel size of 5 px, on subtomograms with a box size of 256 px and a pixel size of 2.55 Å, which were low-pass–filtered to 12 Å. Regions of the occupancy map were masked around the individual E-protein ectodomains (3-Å radius), and the average value of the occupancy inside the mask was calculated. In parallel, the mean intensities of the cryo-EM density values inside the masked volume of the E-protein ectodomains (3-Å radius) were calculated. The mean values of the measured intensities were normalized per each analyzed particle, to represent values in the range of 0 to 1. All the mean occupancy and mean intensity calculations were performed using custom-made scripts and UCSF ChimeraX (*70*). The molecular surfaces of the E-protein ectodomains were colored according to the resulting mean occupancy or normalized mean intensity and displayed using UCSF ChimeraX (*70*).

### Tomogram reconstruction

Movies of individual tilts from the tomographic tilt series were aligned using motion correction implemented in Relion 5.0 beta 3 (*72*). The initial estimation of the CTF of the separate tilt images was done with CtfFind4 software. The tilt series were inspected for dark and ice-contaminated tilt images, to remove them before further processing. The curated tilt series were automatically aligned using IMOD (*73*) patch tracking–based alignment. For template matching, 4× or 8× binned tomograms were reconstructed using Relion 5.0 beta 3 (*72*). Denoised tomograms were reconstructed using CryoCare (*74*) denoiser using noise model trained on the dataset.

### Template matching of immature spikes in tomograms

For template matching of the immature TBEV spike subtomogram locations in the tomograms, pytom-match-pick software was used [https://github.com/SBC-Utrecht/pytom-match-pick; (*75*)]. The template matching was performed on 8× binned, nondenoised, CTF-corrected tomograms, applying a 24-Å low-pass filter for the matching process and featuring the randomized-phase correction (*76*). The coordinates of peaks in the score map above a user-defined threshold were extracted. To filter out false positives, geometry-based (i.e., spatial distance and angular distance between neighboring subtomogram positions) restrictions were applied to the extracted particle locations. The template-matched coordinates and orientations of the matched particles were used for further analysis of subtomogram averaging, data visualization, or counting of the spikes per immature particles.

### Template matching of spikes and herringbone rafts into TBEV maturation intermediates

The maturation intermediates were located in minimum-intensity projection images of cryo-tomograms of TBEV particles along the *z* axis. In total, 27 maturation intermediates were identified in 193 tomograms. The centers of the selected particles were user-defined prior to template matching on 4× binned tomograms in the volume containing the maturation intermediate. The template matching omitted the randomized-phase correction as it produced nonreproducible results for the mature parts of the maturation intermediates. Immature spikes and mature herringbone rafts (three dimers of E-protein ectodomains) extracted from the maps of immature of mature TBEV particles served as search templates. Because of the smooth nature of the herringbone rafts, the template-matching procedure performed well only on the parts of particles perpendicular to the tomogram *z* axis (angular distance of 30°). The results of the template matching were hand-curated, and false positives not agreeing with the particle geometry (e.g., herringbone rafts not located at the particle surface) were removed. To verify the relative alignments of the matched spike and herringbone rafts, whole, icosahedrally symmetrized, immature and mature particles were used as templates for the template matching. Overlay of the results indicated that the orientations of the individually matched spikes and the herringbone rafts followed the icosahedral ordering. For analysis and visualization of the results, the volumes of individual spikes, herringbone rafts, or whole particles were placed back into the tomogram volumes using custom scripts (placeback_subvolume.py; https://github.com/fuzikt/tomostarpy).

### Display of relative orientations of icosahedral symmetries of immature and mature regions in TBEV maturation intermediates

The following procedure was applied to each of the maturation intermediates: The immature domain of the maturation intermediate was rotated to a standard icosahedral orientation (*77*). The rotation to bring the immature domain to the standard orientation was applied to the rotation describing the orientation of the mature domain. The resulting rotated orientation of the mature domain was icosahedrally expanded and one of the 60 icosahedrally related rotations falling within an arbitrarily selected icosahedral asymmetric unit was selected. The selected rotations describing the orientations of the mature domains within the maturation intermediates were used for visualization.

### Statistical evaluation of similarities of the rotations relating icosahedral symmetries of immature and mature domains in TBEV maturation intermediates

Monte Carlo analysis was used to determine whether the relative orientations of immature and mature domains of TBEV maturation intermediates are similar among particles. In case the relative orientations of the domains are similar among particles, the angular distances between the sets of rotation angles describing the relative orientations of the immature and mature domains must be substantially smaller than those of sets of random rotations. Because the immature and mature domains are icosahedrally arranged, the angular distances were analyzed considering the 60 symmetry-related variants of each domain orientation. Therefore, the mean shortest icosahedrally restricted angular distance among the rotation angles relating the immature and the mature domains of the 27 analyzed maturation intermediates was calculated, using the following procedure: (i) The orientation of the immature domain of each maturation intermediate was brought to a standard icosahedral orientation (*77*), and the rotation angles describing the orientation of the mature domain were adjusted accordingly. (ii) The rotation angles, describing the orientation of the mature domain relative to the immature domain in the standard orientation, were used to calculate the angular distance of the orientations of the mature domains among all possible pairs of the maturation intermediates. The procedure considered the 60 icosahedrally equivalent orientations for each pair of maturation intermediates and selected the smallest angular difference. The smallest icosahedrally restricted angular distances from all possible pairwise comparisons were averaged to obtain the mean shortest icosahedrally restricted angular distance [*D*_obs_]. (iii) For the Monte Carlo simulation, sets of 27 random rotation angles from the angular sampling space used in the template matching of the immature and mature structures were selected. The mean shortest icosahedrally restricted angular distance was calculated, as described for the experimental results in steps (i) and (ii) above, for each random set [*D*_rnd_]. (iv) In total, 10,000 random sets were selected. If the shortest icosahedrally restricted angular distance of a randomly selected set is smaller than that of the measured data, then the randomly selected values represent tighter clustering of the orientations than that of the experimental values. Therefore, the *P* value, calculated as *P* = (1 + nr of [*D*_rnd_ ≤ *D*_obs_])/(10,000 + 1), represents the significance of the clustering of the relative orientations of mature domains within TBEV maturation intermediates. The resulting *P* = 0.76 indicates that there is no significant relationship among the relative orientations of immature and mature domains of TBEV maturation intermediates.

### Determination of the number of spikes per immature TBEV particle

Template matching of prM-E spike structure into tomograms was used to determine the number of spikes in each immature particle. The template matching was performed as described above for the analysis of the asymmetric reconstructions of immature TBEV particles. In parallel, template matching of whole immature TBEV particles was performed and manually curated to remove particles on carbon or adjacent to the carbon support and particles close to the tomogram edges. To remove false positives from the template matching of prM-E spikes, only prM-E spikes located less than 300 Å from the centers of particles positioned by the independent template-matching search were included in further analysis. The spikes were clustered around particle centers, and their coordinates were used for least squares fitting of a spherical surface. The spikes positioned more than two standard deviations from the fitted spherical surface were discarded. This resulted in 2990 clusters (each representing an immature particle), while the number of spike coordinates per cluster indicated the spike coverage per immature particle. The analysis of the number of spikes-per-particle was plotted using a violin plot combined with box plot using the programs R and ggplot2 (*78*). Custom-written Python scripts using components from the tomostarpy package were used for analysis of the spike coverage of individual particles (https://github.com/fuzikt/tomostarpy).

### Subtomogram averaging of prM-E spikes

After extraction of 6× binned (pixel size: 8.1 Å), template-matched, subtomograms containing prM-E spikes, subtomogram reconstruction was performed using the orientations estimated during the template matching. The resulting low-resolution initial model was refined at 6× binning reaching the Nyquist frequency and was extracted at 3× binning (pixel size: 4.03 Å) followed by 3D refinement reaching 9.6-Å resolution using the program Relion 5.0 (*62*). The resulting particles were re-extracted in boxes of 192 px without binning, and polishing was performed while only the global tilt positions were polished (without polishing the individual particle positions) followed by defocus refinement improving the resolution to 6.8 Å. Another round of particle position polishing and defocus refinement improved the resolution to 5.0 Å. This was followed by 3D refinement of particles where a segment mask was applied that included only the signal from the immature TBEV spike and the parts of E proteins from neighbors underneath the spike (similar to the mask applied in single particle analysis (SPA) reconstruction of the prM-E spike). After additional polishing and defocus refinement, the resolution improved to 4.3 Å. The final 3D refinement engaged the Blush regularization, and after the final polishing and defocus refinement step, the final FSC_0.143_ resolution reached 4.2 Å.

### Heterogeneity analysis of the subtomograms of immature spikes

The extracted subtomograms of prM-E spikes identified by template matching at binning 6 (box size: 48; pixel size: 8.08 Å) were classified into 30 classes to remove bad particles using Relion 5.0 beta 3 (*72*). The good particles were further 3D-classified into 50 classes. To keep the focus on the low-resolution features, a regularization factor of 0.1 was used. After 75 iterations, 28 classes were occupied (fig. S7), showing the spike heterogeneity.

### Data visualization

UCSF ChimeraX was used for the visualization of all maps and molecular models (*70*). FIJI/ImageJ was used for the visualization of slices of maps and slices of tomograms (*79*). The spherical projection of particle map intensity shells was prepared using a custom-made script mrcpy_spherical_shell_mapper.py (https://github.com/fuzikt/mrcpy).

### Molecular dynamics simulations of membrane bending

#### System preparation

To set up molecular dynamics simulations, we used the PDB structures of mature (PDB: 5O6A) and immature (PDB: 8PUV) TBEV particles (*7*, *13*). The missing side chains of residues of transmembrane helices of the immature TBEV structure were simulated using PDBFixer (*80*). The reconstructed structure was energy-minimized in OpenMM (*81*) with CHARMM36m parameters (*82*), applying a 1000 kJ/mol energy tolerance and positional restraints on the original atoms with the force constant of 5000 kJ/mol/nm^2^. Membrane-associated regions were specifically analyzed for their ability to bend membranes. In both immature and mature virions, perimembrane (PM) helices (chains A, B, and C; residues 398 to 452) and transmembrane (TM) helices (chains A, B, and C; residues 452 to 492) of protein E were included. In the immature virion, the PM (chains D, E, and F; residues 111 to 127) and TM helices (chains D, E, and F; residues 127 to 160) of protein prM were analyzed. For the mature virion, analysis focused on the PM (chains D, E, and F; residues 22 to 39) and the TM helices (chains D, E, and F; residues 39 to 72) of protein M.

The atomistic models were energy-minimized in vacuum using CHARMM36m parameters in GROMACS (version 2022.3) (*82*), with a particle force tolerance of 100 kJ/mol/nm. After minimization, the structures were coarse-grained with Martinize2 (*83*), using Martini v2.2 parameters (*84*). Cryo-EM structures were used to assign secondary structure elements using the program DSSP (*85*). To preserve the tertiary structure, an elastic network model was applied with a 500 kJ/mol/nm^2^ force constant with lower and upper cutoffs of 0.0 and 0.9 nm, respectively. Protein termini were treated as charged, with histidine residues assumed to be in a neutral protonation state unless specified otherwise.

To study protein-lipid interactions and membrane bending, the perimembrane or transmembrane helices were inserted into a pure 1-palmitoyl-2-oleoyl-sn-glycero-3-phosphocholine bilayer using the Insane tool (*86*), solvated by Martini water beads, and ions were added to neutralize the system and obtain 0.15 M NaCl concentration. To prevent freezing, 10% of water beads were substituted by antifreeze water beads. The simulation box dimensions were ~14 nm by 14 nm by 9 nm, and periodic boundary conditions were applied in all directions. In addition, larger membrane systems (20 nm by 20 nm by 15 nm) with three copies of the prM-E or M-E heterodimers were simulated to evaluate membrane deformation as a function of the relative configuration of membrane-associated helices. In this case, an ER membrane mimic (55 mol % 1-palmitoyl-2-oleoyl-*sn*-glycero-3-phosphocholine, 25 mol % 1-palmitoyl-2-oleoyl-*sn*-glycero-3-phosphoethanolamine, 10 mol % 1-palmitoyl-2-oleoyl-*sn*-glycero-3-phosphoinositol, and 10 mol % 1-palmitoyl-2-oleoyl-*sn*-glycero-3-phospho-l-serine) was prepared and solvated as described above. This setup was used to examine the clustering of specific lipid species around the protein and their effect on membrane bending. To retain the orientations and positions from the PDB structures, positional restraints were applied to the protein backbone beads with a force constant of 3000 kJ/mol/nm^2^.

#### Simulation parameters

All systems were first energy-minimized using a steep descent algorithm with a particle force tolerance of 100 kJ/mol/nm. Subsequently, they were equilibrated for 8 μs using the standard Martini v2.2 new-rf simulation parameters (*87*). The Verlet integrator with a 20-fs timestep was used. Short-range interactions were cut off at 1.1 nm using a shifted potential, while long-range interactions were treated using a reaction field (relative permittivity = 15). The temperature was maintained at 310 K via stochastic velocity rescaling (*88*) (coupling constant = 1.0 ps), applied separately to the protein, membrane, and solvent. The pressure was controlled at 1 bar using a semi-isotropic Parrinello-Rahman barostat (*89*) with a coupling constant of 12.0 ps and with the compressibility set to 3 × 10^−4^ bar^−1^. The equilibration was followed by a 10-μs production simulation with identical settings.

#### Analysis

Only the last 1 μs of the trajectory was used for analysis. Membrane curvature and thickness were analyzed using the g_lomepro tool (*90*). The simulation box was divided into 50 by 50 bins for systems containing PM or TM helices of proteins and 100 by 100 bins for the trimeric systems. The high curvature modes (above 1 nm^−1^), corresponding to fluctuations, were filtered out as suggested by the developers (*90*).
