# Supplementary material for: Structures of asymmetric particles of tick-borne encephalitis virus provide insight into flavivirus assembly and maturation
Source: Sci Adv. 2026 Jul 3;12(27):eaee4765. doi: 10.1126/sciadv.aee4765 (PMC13330823; doi:10.1126/sciadv.aee4765)
Supplement: Supplementary file 1 — Figs. S1 to S17 Table S1 [file sciadv.aee4765_sm.pdf]

Science Advances
AAAS

Supplementary Materials for

Structures of asymmetric particles of tick-borne encephalitis virus provide insight into flavivirus assembly and maturation

Tibor Füzik *et al.*

Corresponding author: Sarah J. Butcher, sarah.butcher@helsinki.fi; Pavel Plevka, pavel.plevka@ceitec.muni.cz

Sci. Adv. **12**, eaee4765 (2026)
DOI: 10.1126/sciadv.aee4765

This PDF file includes:

Figs. S1 to S17
Table S1

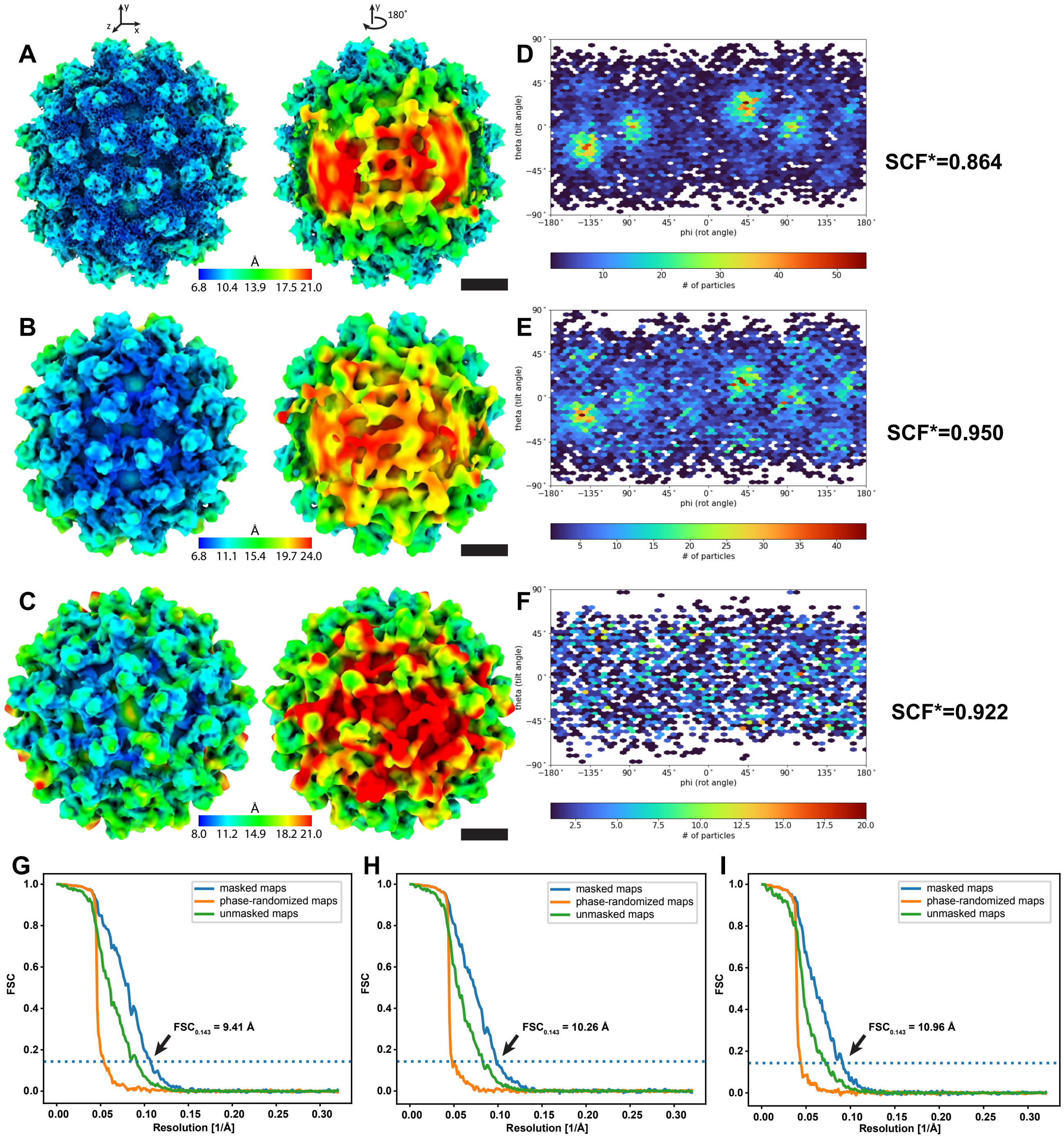

Fig. S1. Resolution and quality of the reconstructions of asymmetric immature TBEV particles. (ABC) Surface representations of local resolution maps colored according to the resolution. Opposite hemispheres of each reconstruction are shown. Scalebars represent 10 nm. **(DEF)** Polar plots of orientations of particle images contributing to the reconstruction. The sampling compensation factor (SCF*) higher than 0.81 indicates that the reconstruction is not affected by preferential particle orientations. **(GHI)** Fourier shell correlation (FSC) curves of masked FSC corrected half-maps (blue), unmasked half-maps (green), and phase randomized masked half-maps (red) of individual cryo-EM reconstructions. The final resolution is reported for the FSC cutoff at 0.143. Dashed line marks FSC cutoff.

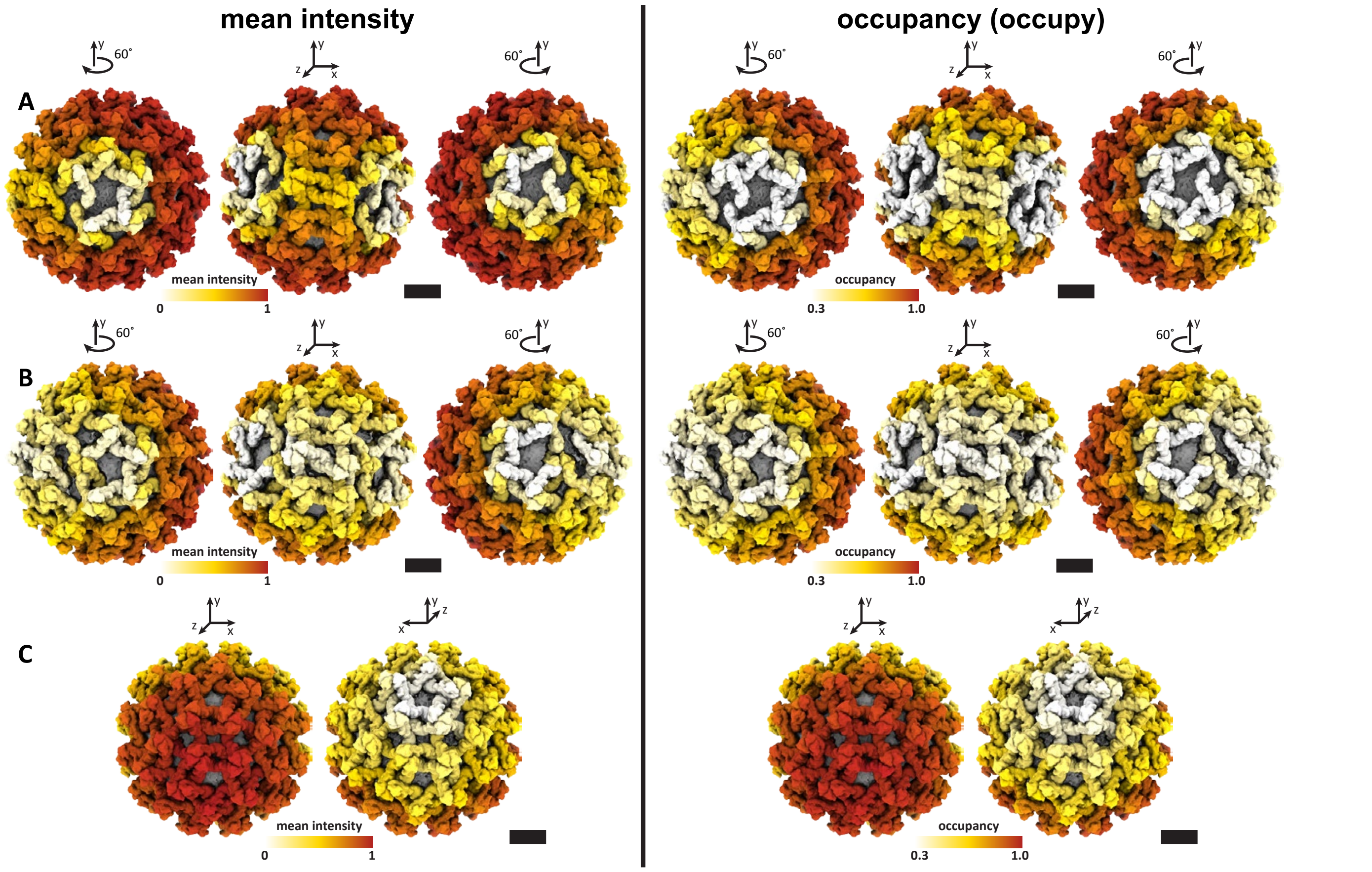

Fig. S2. Occupancy analysis of classes from single particle analysis of TBEV immature particles. (AB) Molecular surface representations of E protein ecto-domains colored according to the normalized mean intensity of the map values, and occupancy of the local cryo-EM density distribution. The map occupancy values were calculated by Occupy. The panels **(ABC)** correspond to the classes presented in Fig. 2**(ABC)** in the main text. Scale bars represent 10 nm in all panels.

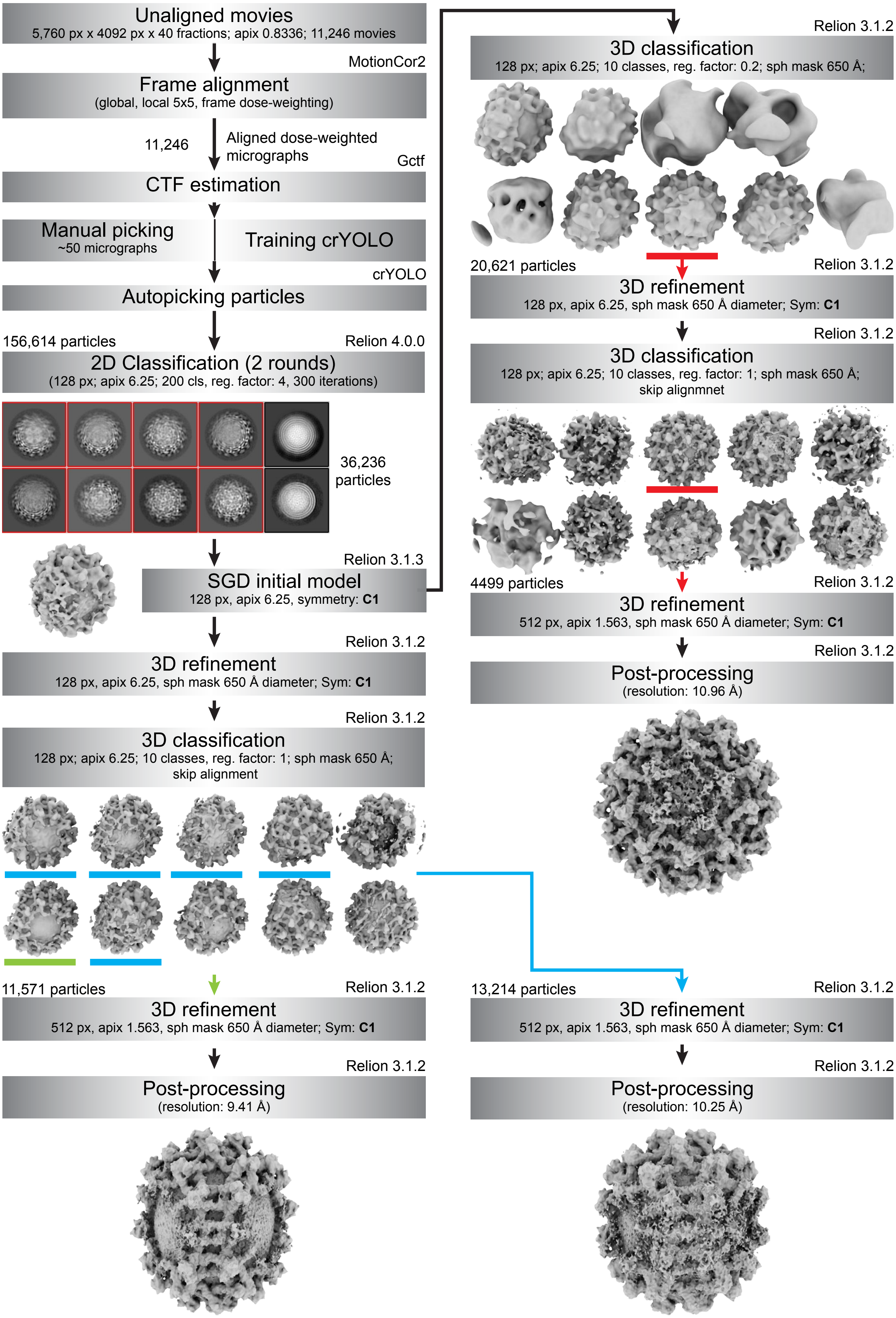

Fig. S3. Scheme of the single particle cryo-EM classification and reconstruction of immature particles of TBEV. Example images of 2D classes are identical to those shown in Fig. S5.

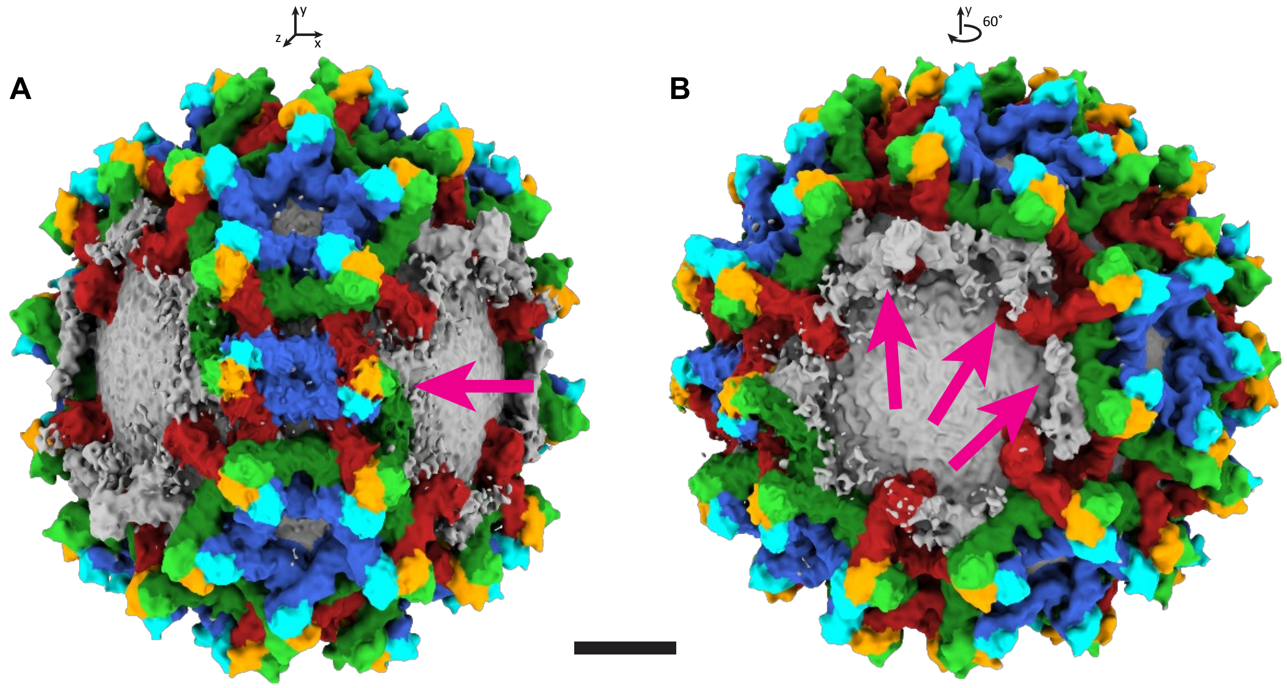

Fig. S4. Structure of asymmetric immature TBEV particle missing two pentamers of prM-E spikes. Surface representation of the cryo-EM density is zone colored based on the fitting of prM-E spikes. E proteins are shown in darker shades of red, blue, and green, while prM proteins are shown in lighter colors. This is the same reconstruction as that shown in Fig. 2**(ADG)**. Pink arrow in panel **(A)** indicates the position of two additional prM-E spikes placed between the exposed membrane areas. Pink arrows in panel **(B)** indicate positions of cryo-EM densities of putative incomplete prM-E spikes. Scale bar represents 10 nm.

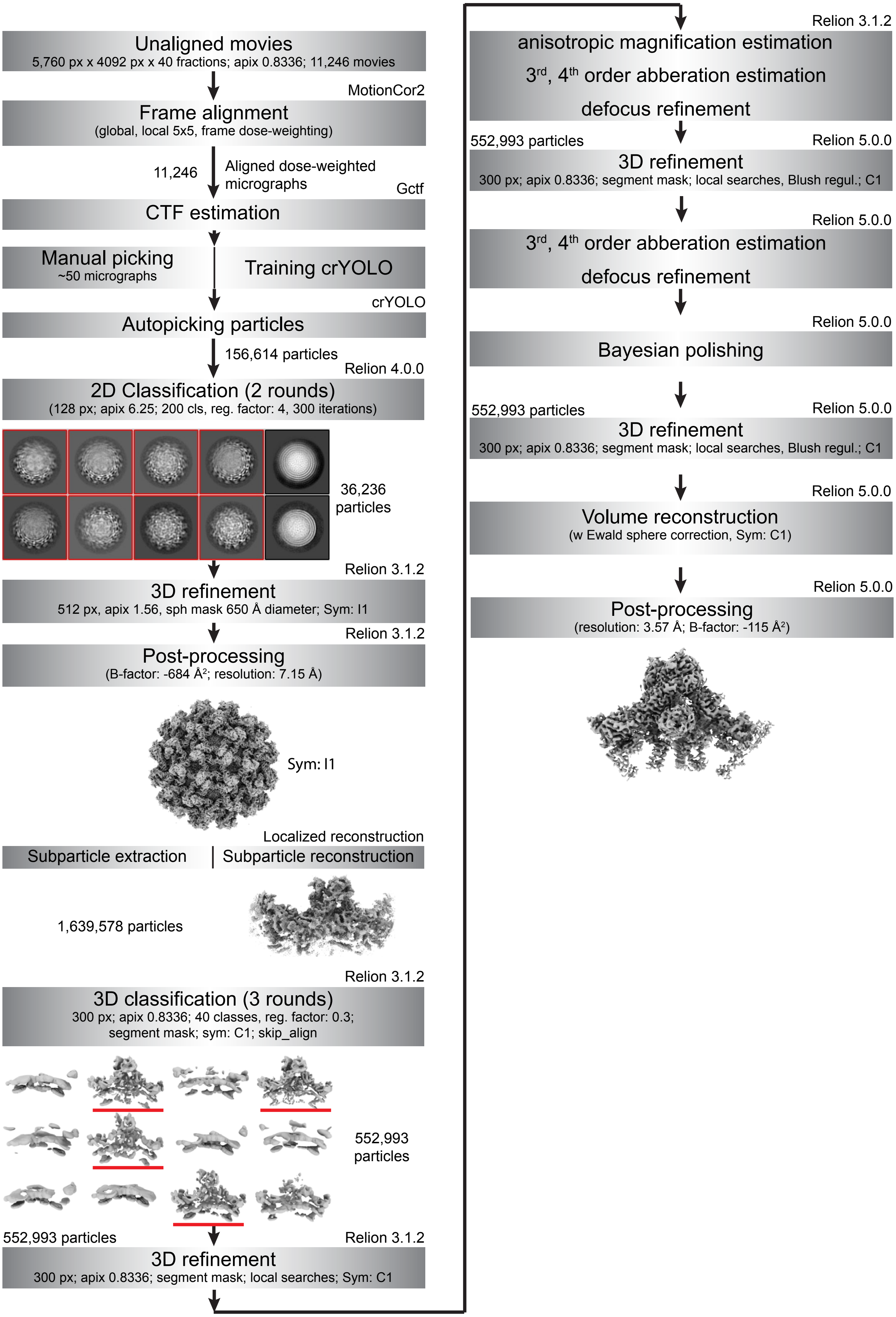

Fig. S5. Scheme of the single particle cryo-EM classification and reconstruction of prM-E spikes of TBEV. Example images of 2D classes are identical to those shown in Fig. S3.

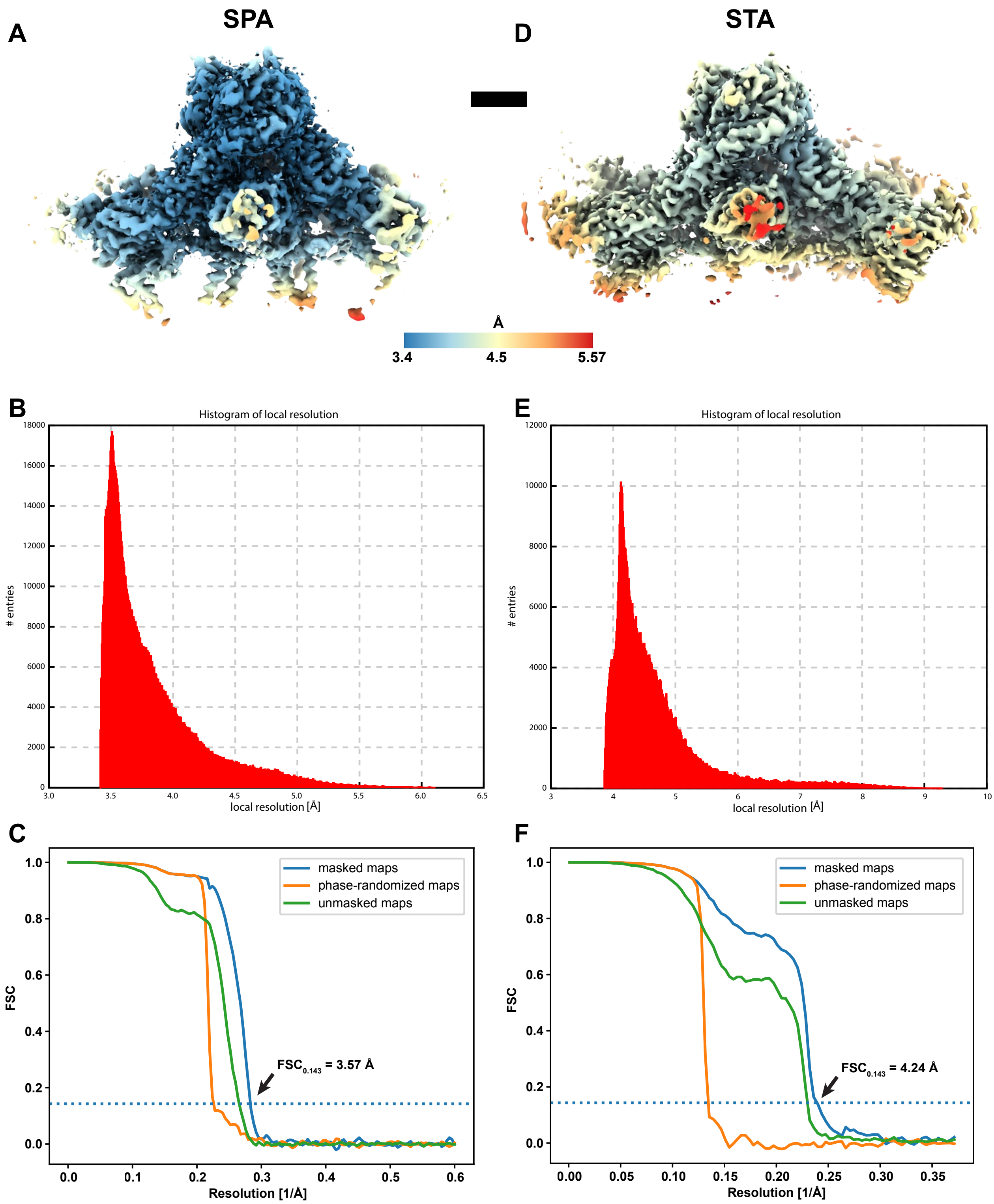

Fig. S6. Cryo-EM and cryo-ET reconstructions of the prM-E spike of TBEV. Single particle reconstruction **(A-C)** and sub-tomogram averaging reconstruction **(D-F)** of prM-E spikes. **(AD)** Surface representations of local resolution maps. The map surfaces are colored according to the local resolution. Scale bar represents 25 Å. **(BE)** Histograms of the distribution of cryo-EM map voxels according to their resolution. **(CF)** Fourier shell correlation (FSC) curves of masked FSC corrected half-maps (blue), unmasked half-maps (green), and phase randomized masked half-maps (red) of individual cryo-EM reconstructions. The final resolution is reported for the FSC cutoff at 0.143. Dashed line marks FSC cutoff.

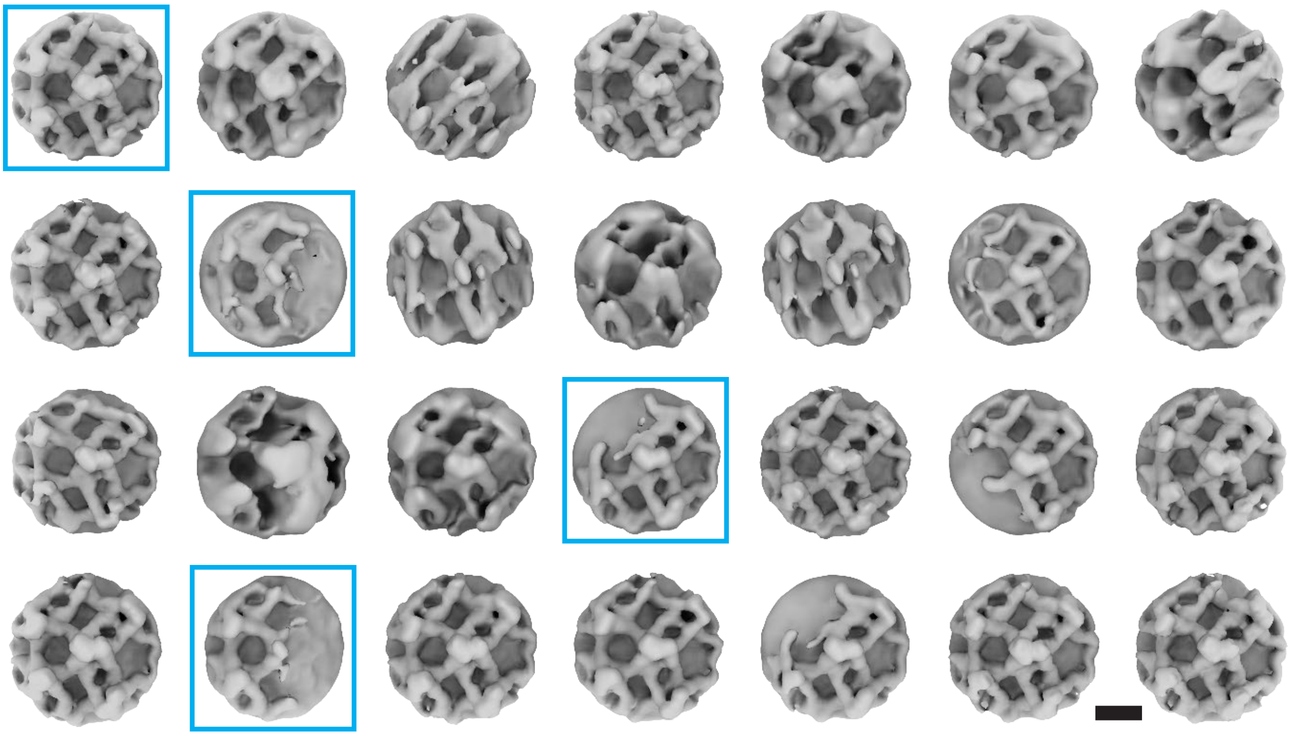

Fig. S7. Three-dimensional class averages of prM-E spikes from tomograms of immature TBEV particles. The classification identified complete and incomplete spikes. The classes highlighted by blue outlines are displayed in Fig. 3. The scale bar represents 5 nm.

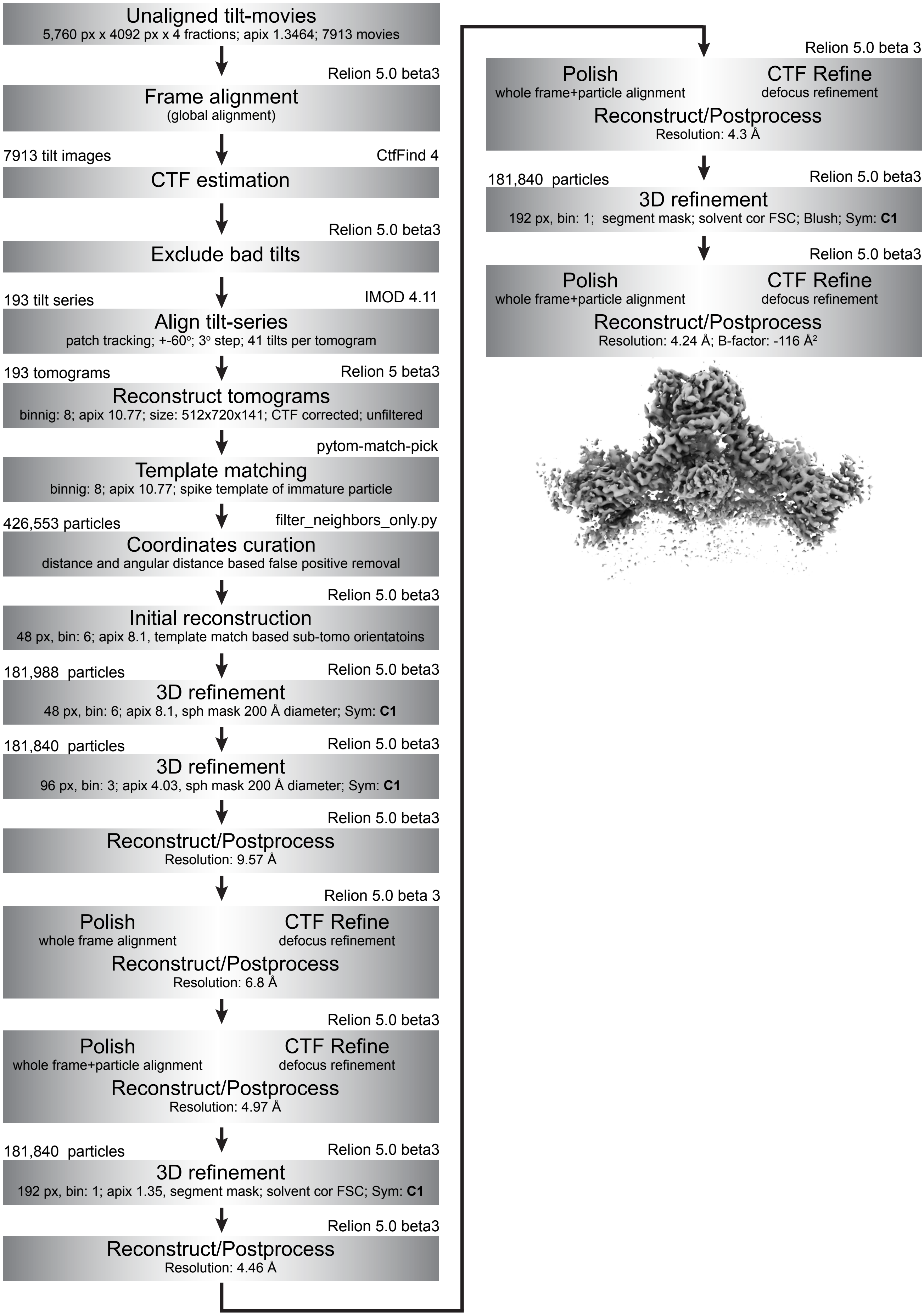

Fig. S8. Scheme of the sub-tomogram classification and averaging of prM-E spikes TBEV.

A

Hydrogen bonds

##	Structure 1	Dist. [Å]	Structure 2
1	G:THR 303[OG1]	2.97	A:THR 56[OG1]
2	G:THR 303[O]	3.25	A:ASN 221[ND2]

No disulfide bonds found
No covalent bonds found
No salt bridges found

Interfacing residues (not a contact table)

Inaccessible residues
Solvent-accessible residues
HSDC Residues making **H**ydrogen/**D**isulphide bond, **S**alt bridge or **C**ovalent link
Interfacing residues
ASA Accessible Surface Area, Å² **BSA** Buried Surface Area, Å² **Δ^iG** Solvation energy effect, kcal/mol |||| Buried area percentage, one bar per 10%

##	Structure 1	HSDC	ASA	BSA	Δ^iG
32	G:THR 32		0.00	0.00	0.00
135	G:GLU 295		120.78	1.72	-0.02
136	G:LYS 296		88.20	18.16	-0.48
138	G:LYS 298		111.76	58.51	0.76
142	G:LEU 302		117.37	6.64	-0.05
143	G:THR 303	H	128.05	95.56	0.51
144	G:TYR 304		57.82	27.78	0.32
145	G:THR 305		88.59	50.60	0.64
175	G:THR 335		119.96	72.24	0.86
176	G:LYS 336		88.82	27.02	-0.24
177	G:PRO 337		54.20	46.86	0.53
178	G:CYS 338		0.00	0.00	0.00
184	G:ALA 344		0.00	0.00	0.00
206	G:ASN 366		107.04	27.07	-0.24
212	G:ILE 372		0.00	0.00	0.00
214	G:MET 374		0.00	0.00	0.00
223	G:ILE 383		0.00	0.00	0.00

##	Structure 2	HSDC	ASA	BSA	Δ^iG
3	A:CYS 3		0.00	0.00	0.00
42	A:ASP 42		0.00	0.00	0.00
56	A:THR 56	H	44.39	25.97	-0.11
57	A:ARG 57		38.32	26.94	-0.70
60	A:CYS 60		0.00	0.00	0.00
80	A:ALA 80		0.00	0.00	0.00
113	A:ILE 113		0.00	0.00	0.00
114	A:VAL 114		0.00	0.00	0.00
115	A:ALA 115		0.00	0.00	0.00
116	A:CYS 116		0.00	0.00	0.00
127	A:ALA 127		0.00	0.00	0.00
129	A:GLY 129		0.00	0.00	0.00
131	A:VAL 131		44.80	18.58	0.30
133	A:ASP 133		52.69	12.53	-0.11
135	A:ASN 135		88.83	27.65	-0.18
136	A:LYS 136		113.14	14.83	-0.50
168	A:SER 168		91.18	13.56	0.22
195	A:ALA 195		66.09	0.84	0.01
198	A:VAL 198		4.32	2.67	0.04
200	A:LEU 200		0.00	0.00	0.00
217	A:ARG 217		87.45	30.88	-0.07
220	A:PHE 220		0.00	0.00	0.00
221	A:ASN 221	H	76.92	37.76	-0.39
227	A:TRP 227		57.93	47.17	0.75
229	A:HIS 229		93.05	68.01	0.30
231	A:GLY 231		64.34	3.50	0.06
232	A:ALA 232		55.30	20.69	0.28
236	A:ASN 236		77.18	40.85	-0.40
237	A:ASN 237		74.28	21.10	-0.19

Hydrogen bonds

##	Structure 1	Dist. [Å]	Structure 2
1	I:LEU 302[N]	3.80	C:ASP 222[OD1]
2	I:THR 303[N]	3.89	C:ASP 222[OD1]
3	I:THR 303[OG1]	2.82	C:ASP 218[O]

No disulfide bonds found
No covalent bonds found
No salt bridges found

Interfacing residues (not a contact table)

Inaccessible residues
Solvent-accessible residues
HSDC Residues making **H**ydrogen/**D**isulphide bond, **S**alt bridge or **C**ovalent link
Interfacing residues
ASA Accessible Surface Area, Å² **BSA** Buried Surface Area, Å² **Δ^iG** Solvation energy effect, kcal/mol |||| Buried area percentage, one bar per 10%

##	Structure 1	HSDC	ASA	BSA	Δ^iG
3	I:CYS 3		0.00	0.00	0.00
32	I:THR 32		0.00	0.00	0.00
42	I:ASP 42		0.00	0.00	0.00
63	I:GLU 144		0.00	0.00	0.00
97	I:GLU 178		111.57	48.12	0.36
138	I:LYS 298		113.88	52.53	0.84
139	I:MET 299		39.74	2.94	-0.03
140	I:LYS 300		70.57	38.61	-0.93
141	I:GLY 301		11.06	10.48	-0.03
142	I:LEU 302	H	107.19	30.03	0.40
143	I:THR 303	H	130.09	69.98	0.48
144	I:TYR 304		46.96	27.10	0.30
165	I:THR 325		0.00	0.00	0.00
166	I:VAL 326		0.00	0.00	0.00
177	I:PRO 337		48.66	25.78	0.41
178	I:CYS 338		0.00	0.00	0.00
184	I:ALA 344		0.00	0.00	0.00
205	I:GLU 365		42.60	10.79	-0.18
206	I:ASN 366		106.39	35.63	-0.16
207	I:ASN 367		138.56	18.54	-0.15
214	I:MET 374		0.00	0.00	0.00

##	Structure 2	HSDC	ASA	BSA	Δ^iG
32	C:THR 32		0.00	0.00	0.00
41	C:MET 41		0.00	0.00	0.00
57	C:ARG 57		36.30	16.47	-0.46
113	C:ILE 113		0.00	0.00	0.00
114	C:VAL 114		0.00	0.00	0.00
115	C:ALA 115		0.00	0.00	0.00
116	C:CYS 116		0.00	0.00	0.00
127	C:ALA 127		0.00	0.00	0.00
129	C:GLY 129		0.00	0.00	0.00
141	C:VAL 141		0.00	0.00	0.00
143	C:VAL 143		0.00	0.00	0.00
182	C:VAL 182		0.00	0.00	0.00
199	C:ILE 199		0.00	0.00	0.00
217	C:ARG 217		90.62	27.55	-0.58
218	C:ASP 218	H	82.57	44.08	0.09
219	C:TRP 219		79.26	25.97	0.39
221	C:ASN 221		56.81	28.56	-0.24
222	C:ASP 222	H	105.35	90.96	0.09
223	C:LEU 223		27.93	1.84	-0.02
224	C:ALA 224		76.88	30.80	0.49
227	C:TRP 227		55.41	20.75	0.31
237	C:ASN 237		59.89	1.76	-0.03
239	C:GLU 239		84.79	22.43	-0.01
240	C:ARG 240		96.99	56.64	-1.15
288	C:VAL 288		0.00	0.00	0.00
290	C:CYS 290		0.00	0.00	0.00
344	C:ALA 344		0.00	0.00	0.00
372	C:ILE 372		0.00	0.00	0.00
374	C:MET 374		0.00	0.00	0.00
383	C:ILE 383		0.00	0.00	0.00
385	C:VAL 385		0.00	0.00	0.00

Hydrogen bonds

##	Structure 1	Dist. [Å]	Structure 2
1	H:GLY 301[N]	3.02	B:ASP 222[OD2]
2	H:LEU 302[N]	3.34	B:ASP 222[OD2]

Salt bridges

##	Structure 1	Dist. [Å]	Structure 2
1	H:LYS 298[NZ]	3.92	B:ASP 218[OD1]

No disulfide bonds found
No covalent bonds found

Interfacing residues (not a contact table)

Inaccessible residues
Solvent-accessible residues
HSDC Residues making **H**ydrogen/**D**isulphide bond, **S**alt bridge or **C**ovalent link
Interfacing residues
ASA Accessible Surface Area, Å² **BSA** Buried Surface Area, Å² **Δ^iG** Solvation energy effect, kcal/mol |||| Buried area percentage, one bar per 10%

##	Structure 1	HSDC	ASA	BSA	Δ^iG
3	H:CYS 3		0.00	0.00	0.00
97	H:GLU 178		99.49	52.40	-0.04
138	H:LYS 298	S	111.28	75.55	0.37
139	H:MET 299		26.72	6.01	-0.07
140	H:LYS 300		64.86	31.89	0.21
141	H:GLY 301	H	9.37	9.37	-0.07
142	H:LEU 302	H	117.29	24.79	0.34
143	H:THR 303		121.38	67.54	0.69
144	H:TYR 304		41.57	24.23	0.28
166	H:VAL 326		0.00	0.00	0.00
177	H:PRO 337		55.36	25.59	0.41
184	H:ALA 344		0.00	0.00	0.00
205	H:GLU 365		63.53	9.07	-0.14
206	H:ASN 366		106.55	48.95	-0.40
212	H:ILE 372		0.00	0.00	0.00
214	H:MET 374		0.00	0.00	0.00
223	H:ILE 383		0.00	0.00	0.00
225	H:VAL 385		0.00	0.00	0.00

##	Structure 2	HSDC	ASA	BSA	Δ^iG
32	B:THR 32		0.00	0.00	0.00
57	B:ARG 57		50.69	19.50	-0.62
113	B:ILE 113		0.00	0.00	0.00
114	B:VAL 114		0.00	0.00	0.00
115	B:ALA 115		0.00	0.00	0.00
116	B:CYS 116		0.00	0.00	0.00
117	B:VAL 117		0.00	0.00	0.00
129	B:GLY 129		0.00	0.00	0.00
144	B:GLU 144		0.00	0.00	0.00
217	B:ARG 217		104.31	33.40	-0.72
218	B:ASP 218	S	85.57	60.23	-0.67
219	B:TRP 219		83.31	24.79	0.40
221	B:ASN 221		54.88	27.75	-0.12
222	B:ASP 222	H	102.36	93.72	-0.11
223	B:LEU 223		37.71	0.78	0.01
224	B:ALA 224		77.08	30.00	0.47
227	B:TRP 227		62.28	16.65	0.18
239	B:GLU 239		99.22	8.95	0.04
240	B:ARG 240		94.03	51.59	-0.82
326	B:VAL 326		0.00	0.00	0.00
328	B:MET 328		0.00	0.00	0.00
344	B:ALA 344		0.00	0.00	0.00
372	B:ILE 372		0.00	0.00	0.00
374	B:MET 374		0.00	0.00	0.00
383	B:ILE 383		0.00	0.00	0.00
385	B:VAL 385		0.00	0.00	0.00

B

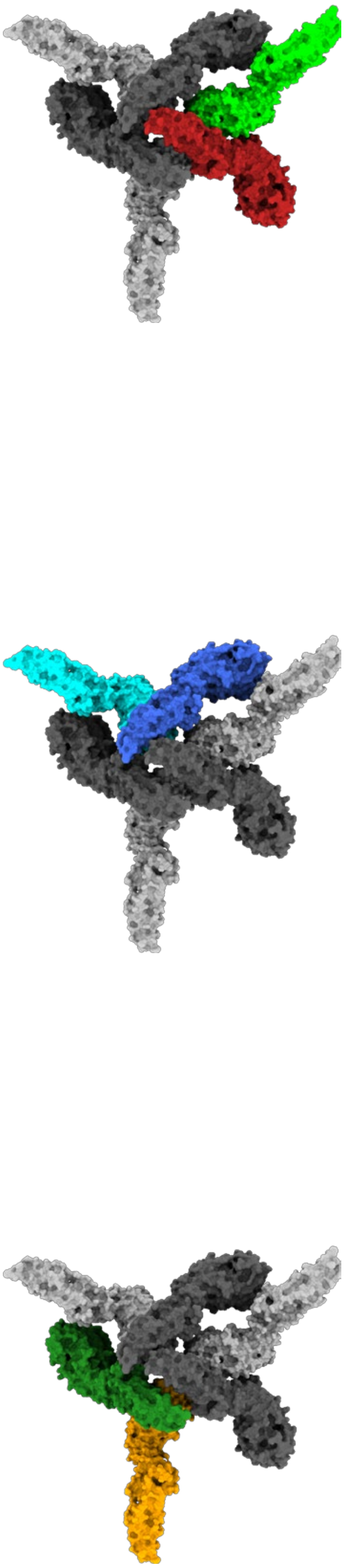

Fig. S9. Interfaces of E proteins from TBEV prM-E spike with E proteins from neighboring spikes. (A) Tables of inter-residue interactions and buried surface areas per residue. **(B)** Molecular surface representation of E proteins from immature spike and their interaction partners from the neighboring spikes. The pairs of analyzed interacting E proteins are shown in color.

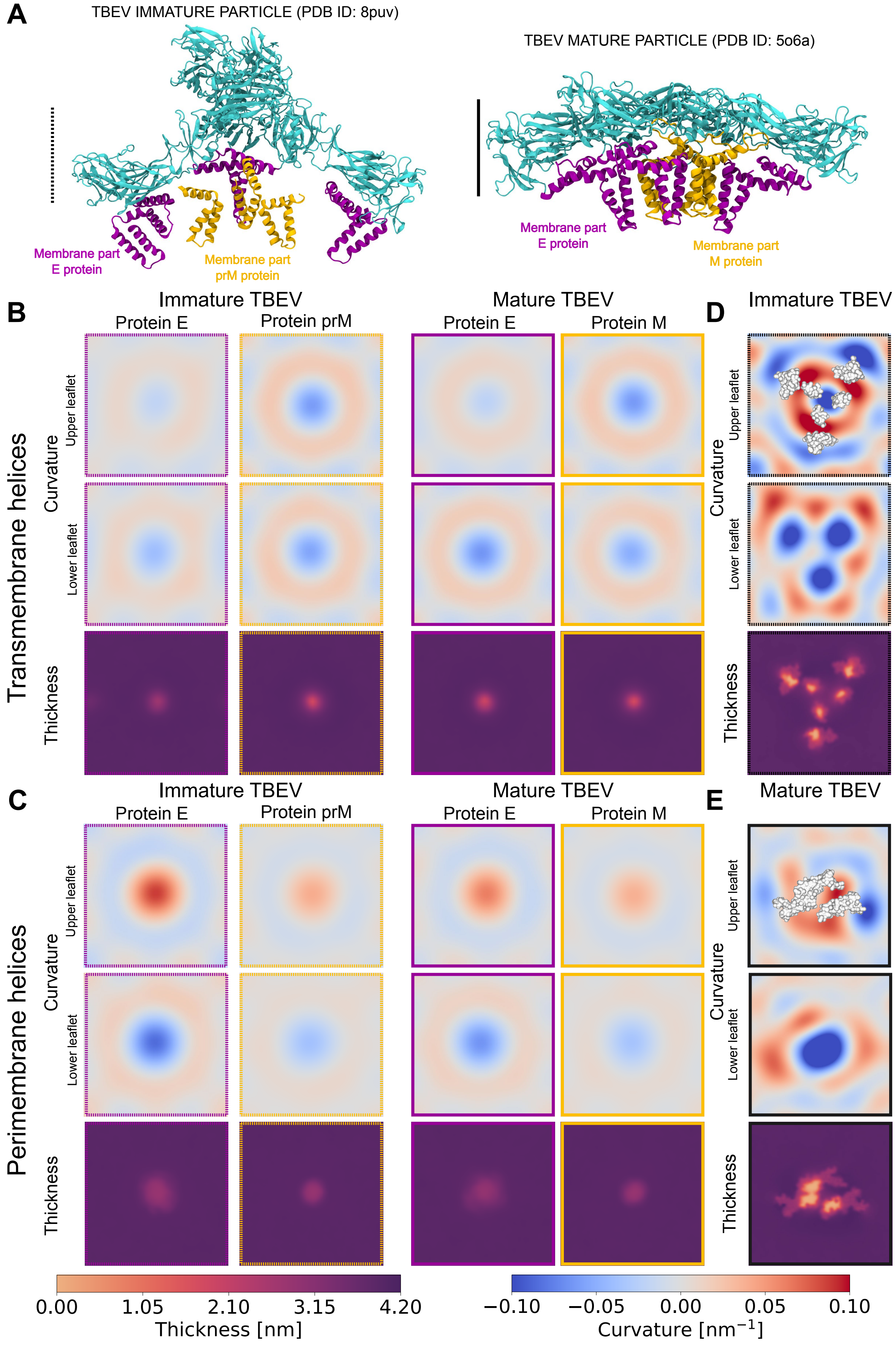

Fig. S10. Trans- and peri-membrane helices of E and prM/M induce membrane bending. Coarse-grained molecular dynamics simulations of the effects of the interactions of trans- and peri-membrane helices of E and prM/M on membrane thickness. **(A)** Cartoon representations of the icosahedral asymmetric unit of TBEV immature particle (left) and virion (right). The membrane helices of E proteins are shown in magenta, prM/M in orange, and the E and prM protein ectodomains are shown in light blue. **(BC)** Effects of transmembrane and perimembrane helices on phosphatidylcholine membrane curvature. The maps are centered on the proteins, which were free to move. **(DE)** Effects of the spatial arrangements of transmembrane and perimembrane helices on the thickness and curvature of an ER-mimicking membrane. The membrane curvature is indicated relative to the membrane bilayer.

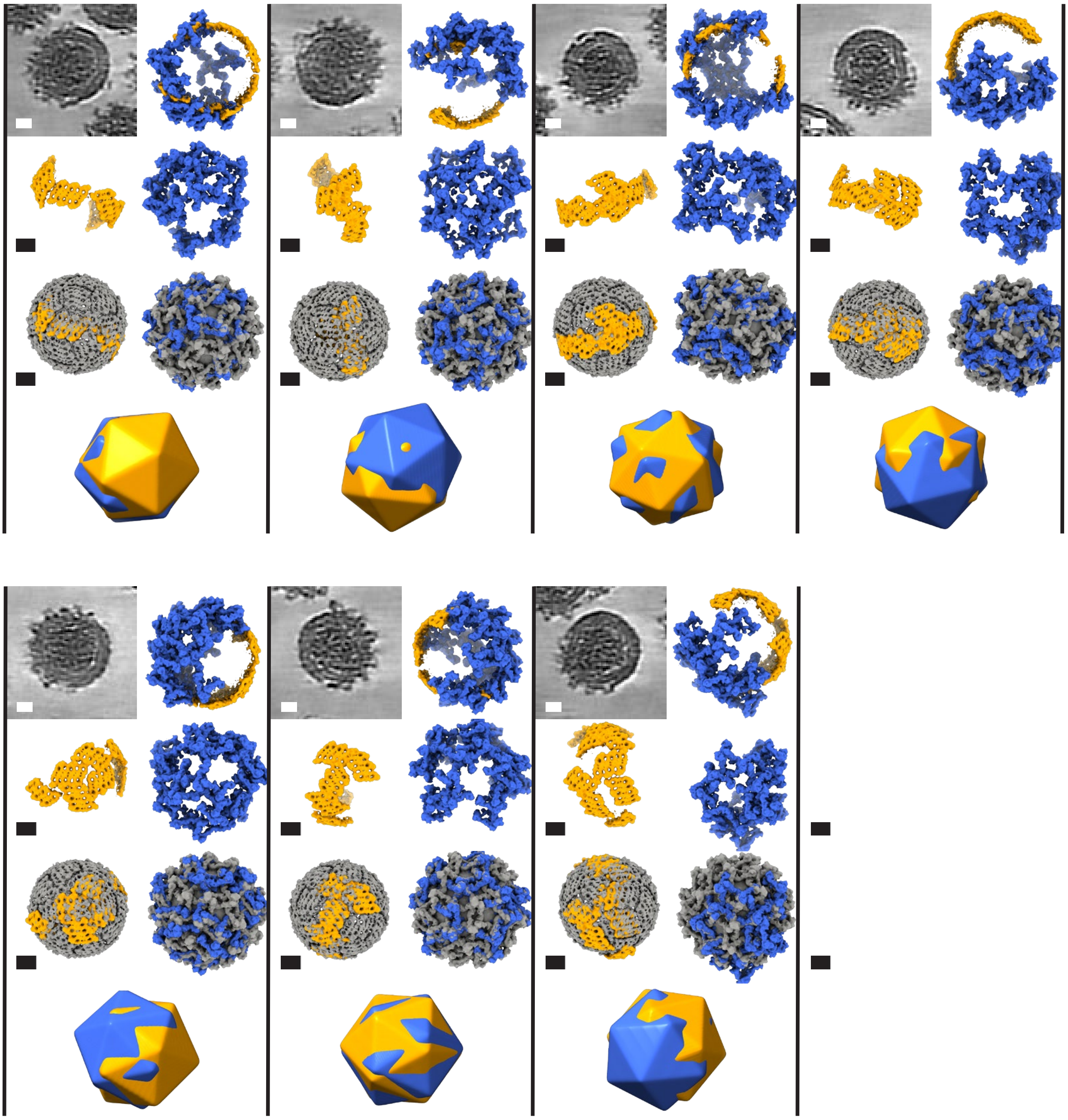

Fig. S11. Template matching analysis of cryo-electron tomographs of TBEV maturation intermediates. Individual panels show: Projection image of a 1.1 nm thick section of cryo-tomogram of a TBEV maturation intermediate (top left). Three-dimensional representation of template fitting of herringbone rafts (orange) and prM-E spikes (blue) into the particle densities (top right). Please note that the search algorithm identified the placement of the herringbone patterns with confidence only when their orientations were approximately perpendicular to the XY plane of the tomogram. Rotated views of the arrangement of herringbone patterns and spikes (second row from the top). Overlays of the individually placed herringbone patterns and spikes with icosahedral mature and immature TBEV structures (gray) (third row from the top). Blue and orange icosahedra show the relative orientations of the icosahedral symmetries of the immature pattern (blue) and mature pattern (orange) at the surfaces of the maturation intermediates (bottom center). Particle images in panels #1 and #3 are identical to the overview of an electron micrograph shown in Fig. 4A. Scale bar represents 10 nm in all panels.

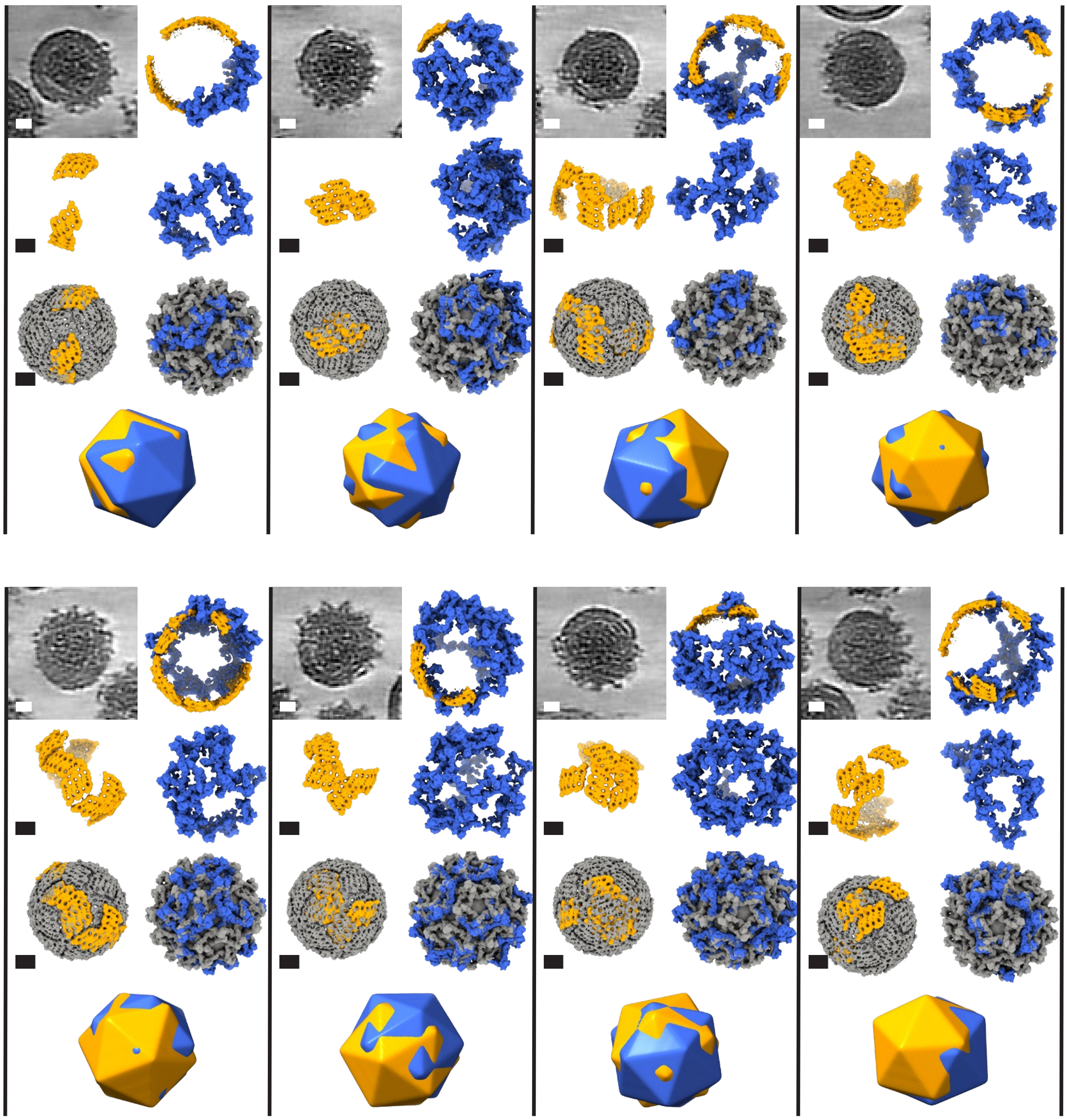

Fig. S11. (continued...) Template matching analysis of cryo-electron tomographs of TBEV maturation intermediates. Individual panels show: Projection image of a 1.1 nm thick section of cryo-tomogram of a TBEV maturation intermediate (top left). Three-dimensional representation of template fitting of herringbone rafts (orange) and prM-E spikes (blue) into the particle densities (top right). Please note that the search algorithm identified the placement of the herringbone patterns with confidence only when their orientations were approximately perpendicular to the XY plane of the tomogram. Rotated views of the arrangement of herringbone patterns and spikes (second row from the top). Overlays of the individually placed herringbone patterns and spikes with icosahedral mature and immature TBEV structures (gray) (third row from the top). Blue and orange icosahedra show the relative orientations of the icosahedral symmetries of the immature pattern (blue) and mature pattern (orange) at the surfaces of the maturation intermediates (bottom center). Scale bar represents 10 nm in all panels.

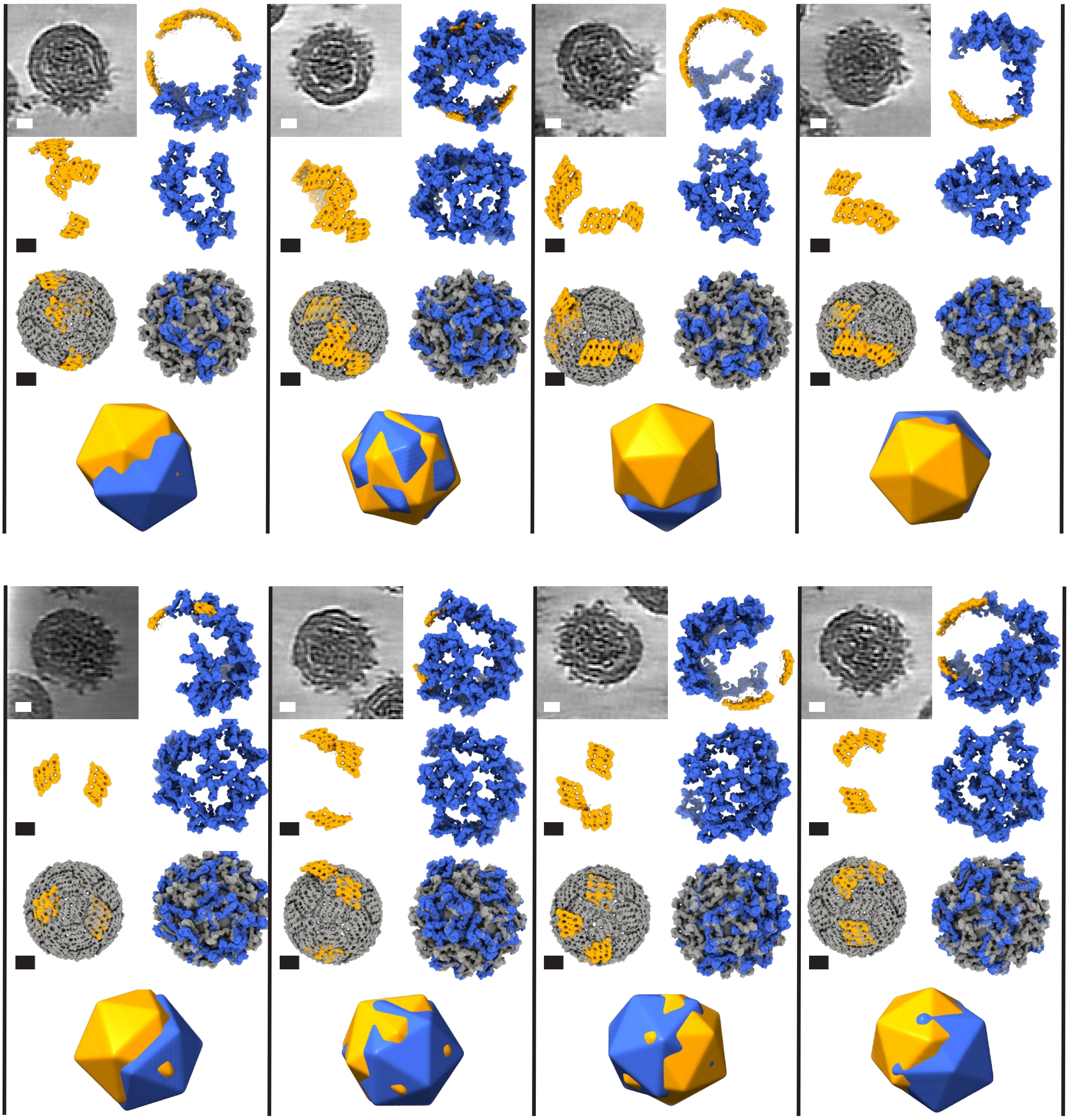

Fig. S11. (continued...) Template matching analysis of cryo-electron tomographs of TBEV maturation intermediates. Individual panels show: Projection image of a 1.1 nm thick section of cryo-tomogram of a TBEV maturation intermediate (top left). Three-dimensional representation of template fitting of herringbone rafts (orange) and prM-E spikes (blue) into the particle densities (top right). Please note that the search algorithm identified the placement of the herringbone patterns with confidence only when their orientations were approximately perpendicular to the XY plane of the tomogram. Rotated views of the arrangement of herringbone patterns and spikes (second row from the top). Overlays of the individually placed herringbone patterns and spikes with icosahedral mature and immature TBEV structures (gray) (third row from the top). Blue and orange icosahedra show the relative orientations of the icosahedral symmetries of the immature pattern (blue) and mature pattern (orange) at the surfaces of the maturation intermediates (bottom center). Scale bar represents 10 nm in all panels.

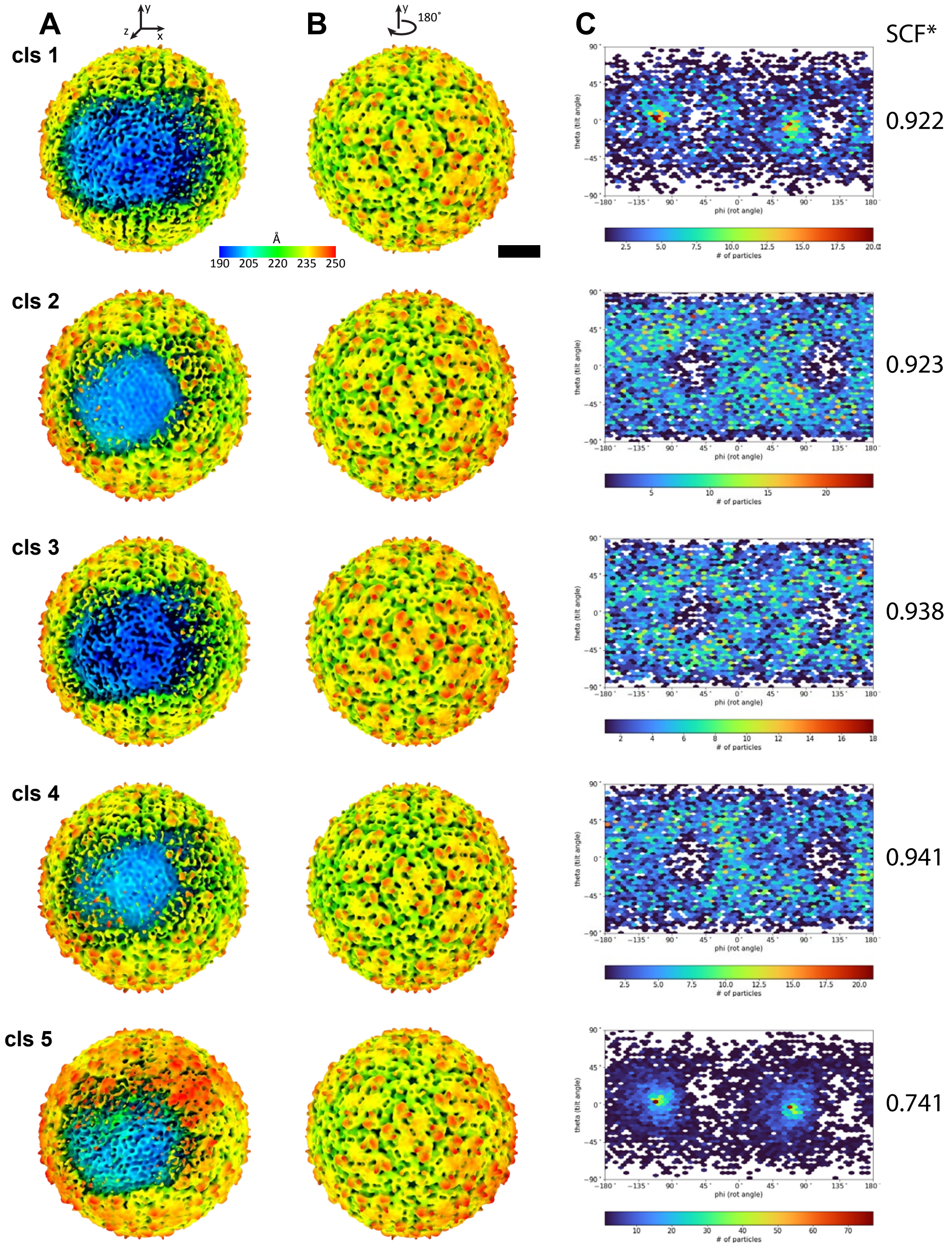

Fig. S12. Classification and single particle analysis of TBEV virions. (AB) Surface representations of cryo-EM maps of asymmetric TBEV virions missing subsets of M-E heterodimers. The maps are rainbow-colored based on the distance from the particle center, ranging from blue to red. The areas of exposed membrane are shown in blue. Opposite hemispheres of each reconstruction are shown in panels **(A)** and **(B)**. **(C)** Polar plots of orientations of particle images contributing to the reconstructions. SCF* higher than 0.81 indicates that the reconstruction is not affected by preferential particle orientations. Scale bars represent 10 nm in all panels.

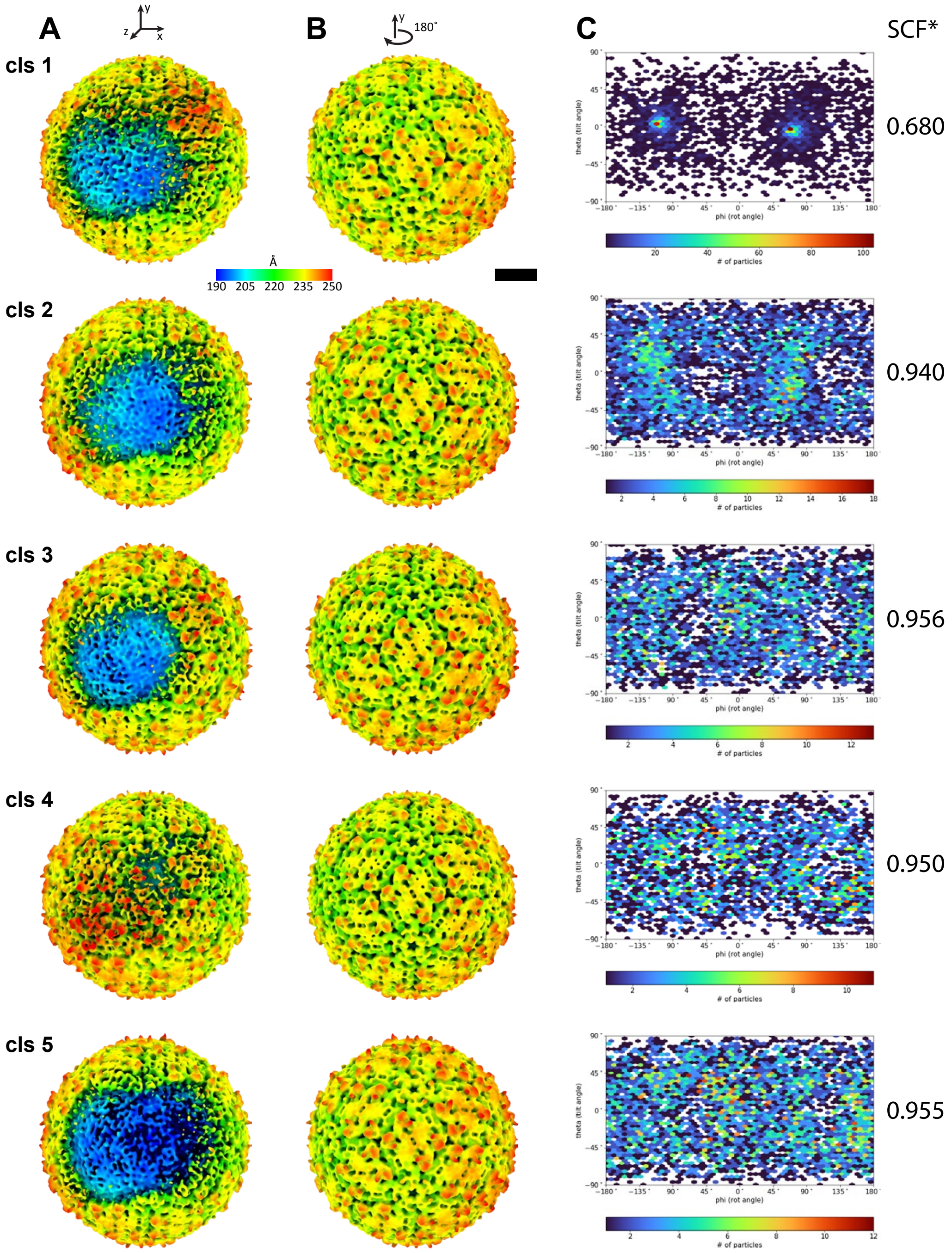

Fig. S12. (continued...) Classification and single particle analysis of TBEV virions. (AB) Surface representations of cryo-EM maps of asymmetric TBEV virions missing subsets of M-E heterodimers. The maps are rainbow-colored based on the distance from the particle center, ranging from blue to red. The areas of exposed membrane are shown in blue. Opposite hemispheres of each reconstruction are shown in panels **(A)** and **(B)**. **(C)** Polar plots of orientations of particle images contributing to the reconstructions. SCF* higher than 0.81 indicates that the reconstruction is not affected by preferential particle orientations. Scale bars represent 10 nm in all panels.

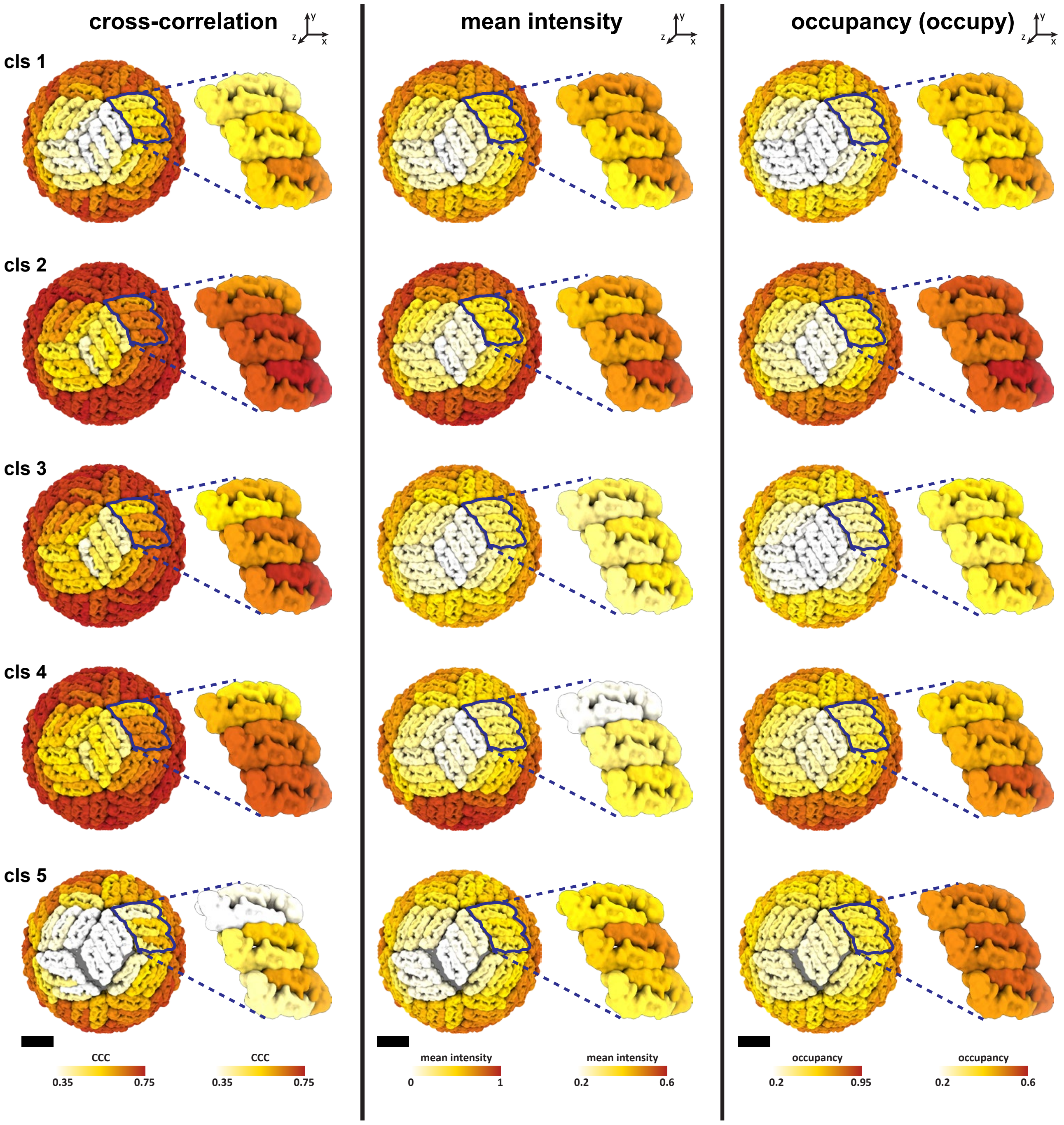

Fig. S13. Occupancy analysis of classes from single particle analysis of TBEV virions. (AB) Molecular surface representations of E protein ecto-domains colored according to the cross-correlation, mean intensity of the map values, and occupancy of the local cryo-EM density distribution. The map occupancy values were calculated by Occupy. Scale bars represent 10 nm in all panels.

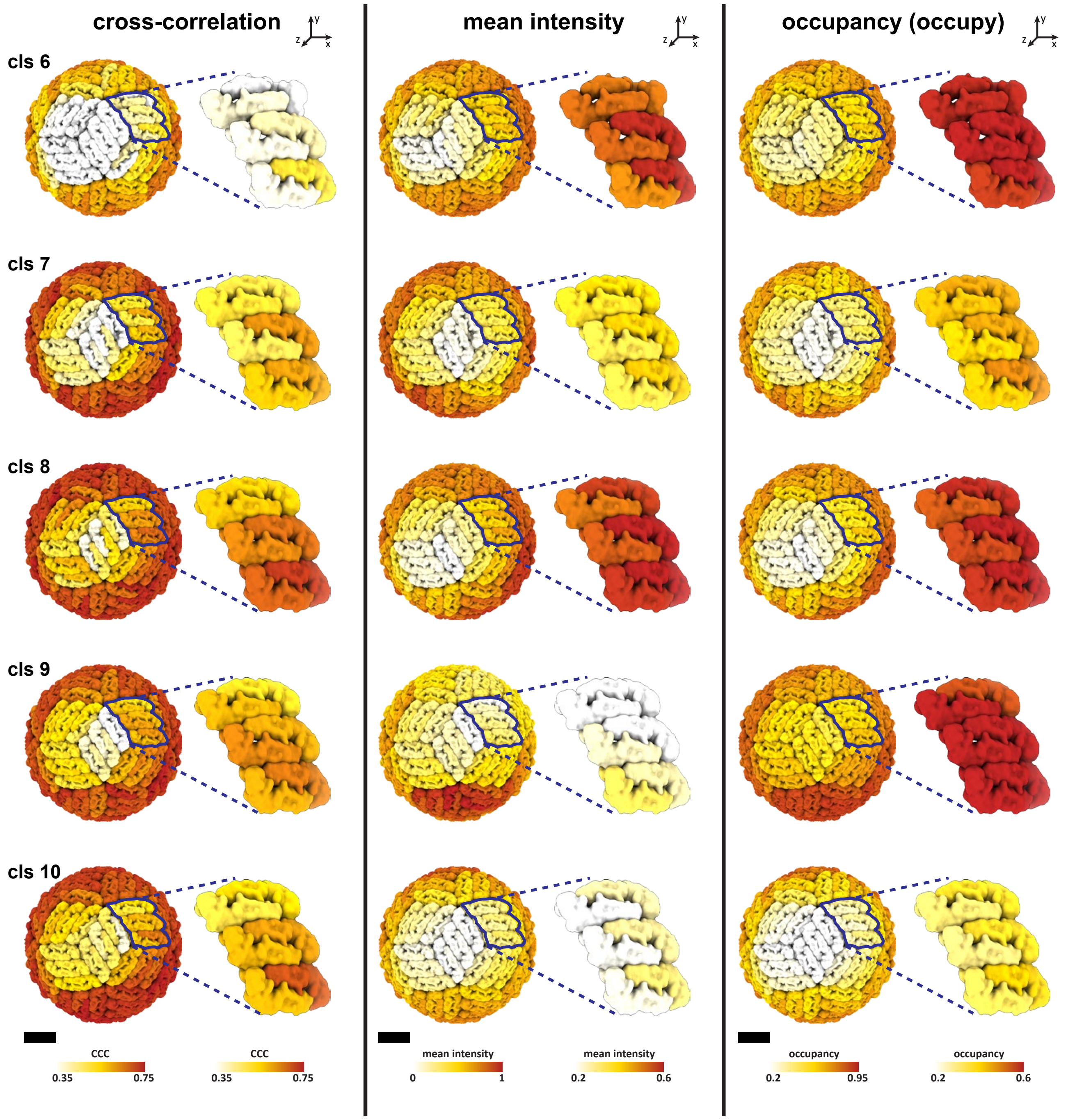

Fig. S13. (continued...) Occupancy analysis of classes from single particle analysis of TBEV virions. (AB) Molecular surface representations of E protein ecto-domains colored according to the cross-correlation, mean intensity of the map values, and occupancy of the local cryo-EM density distribution. The map occupancy values were calculated by Occupy. Scale bars represent 10 nm in all panels.

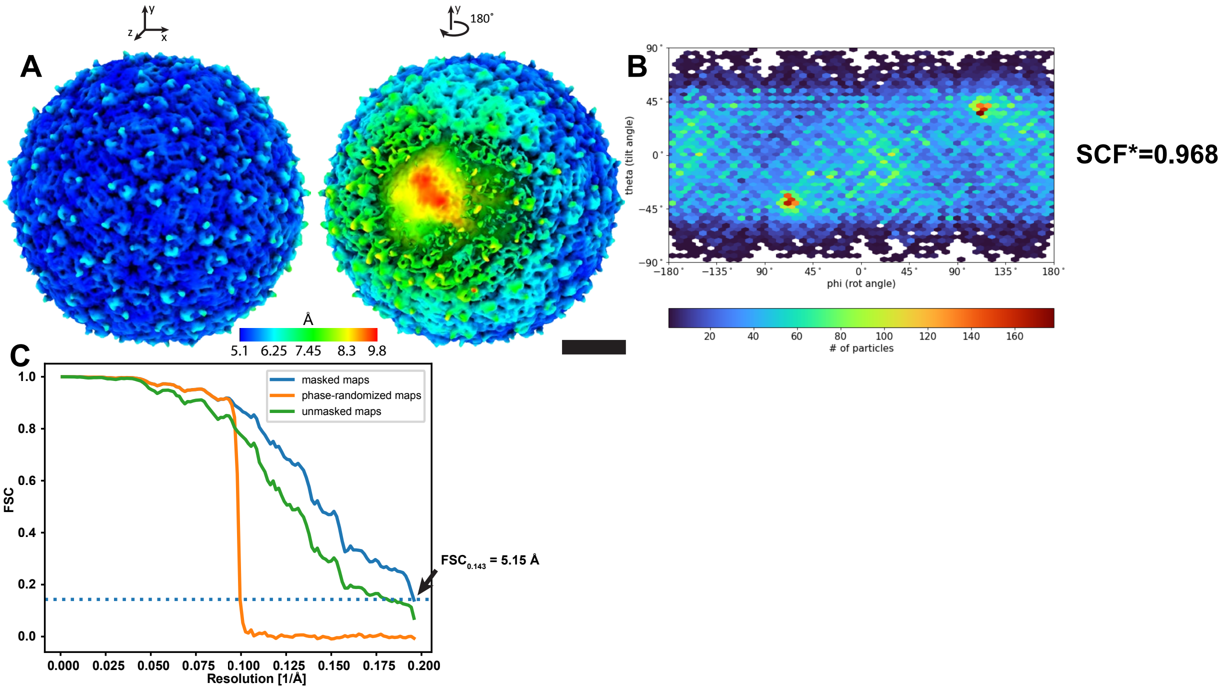

Fig. S14. Resolution and quality of the reconstruction of asymmetric TBEV virion. (A) Local resolution map surface colored according to the resolution. Opposite hemispheres of reconstruction are shown. Scalebar represents 10 nm. **(B)** Polar plot of orientations of particle images contributing to the reconstruction. SCF* higher than 0.81 indicates that the reconstruction is not affected by preferential particle orientations. **(C)** Fourier shell correlation (FSC) curves of masked FSC corrected half-maps (blue), unmasked half-maps (green), and phase randomized masked half-maps (red) of individual cryo-EM reconstructions. The final resolution is reported for the FSC cutoff at 0.143. Dashed line marks FSC cutoff.

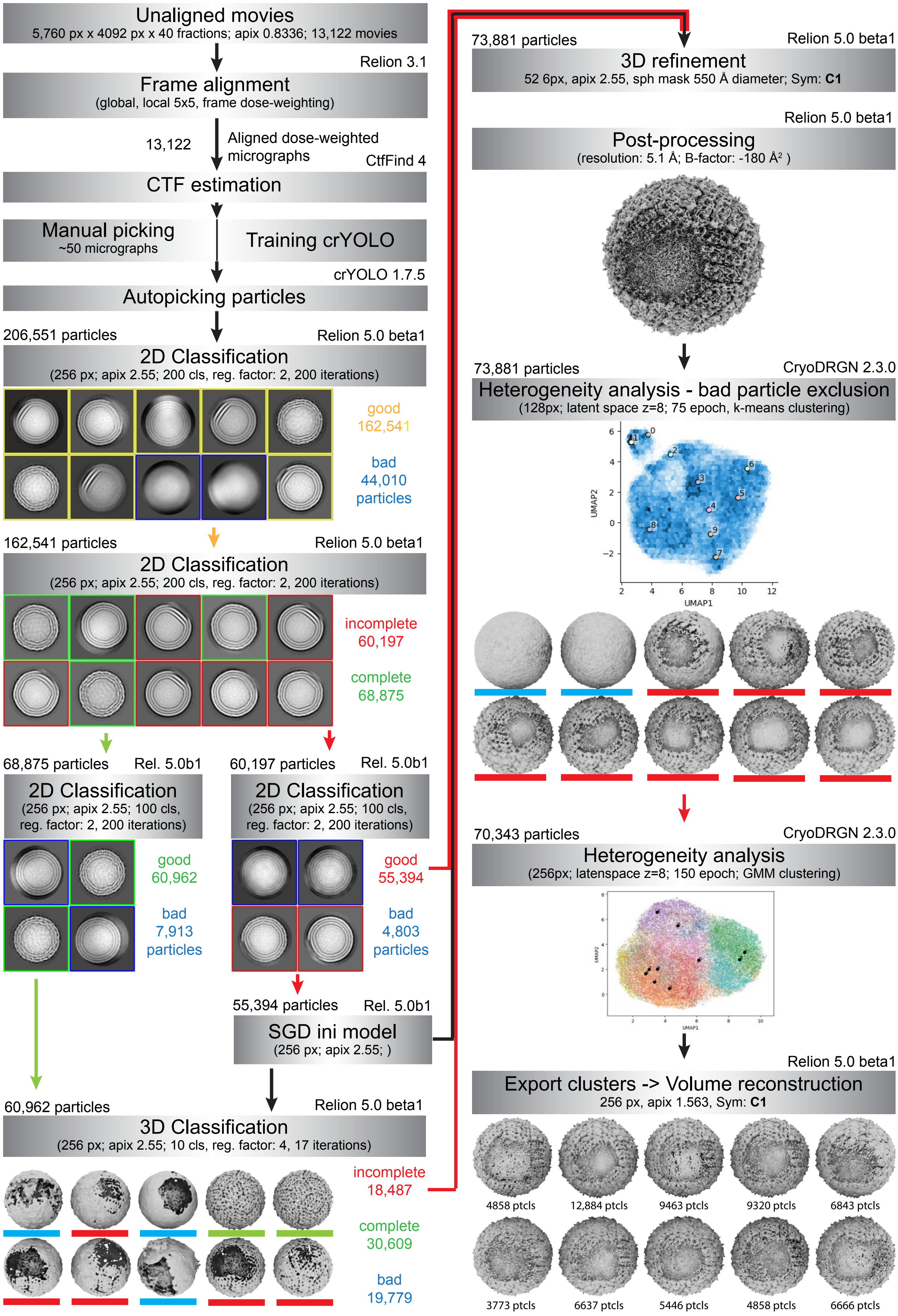

Fig. S15. Scheme of the single particle cryo-EM classification and reconstruction of TBEV virion.

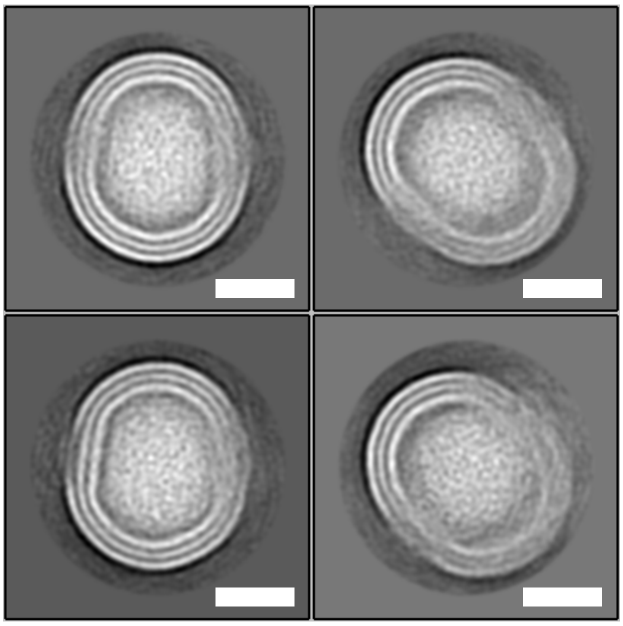

Fig. S16. Two-dimensional class averages of TBEV particles with smooth organization of E proteins that deviate from icosahedral symmetry. All scale bars represent 20 nm.

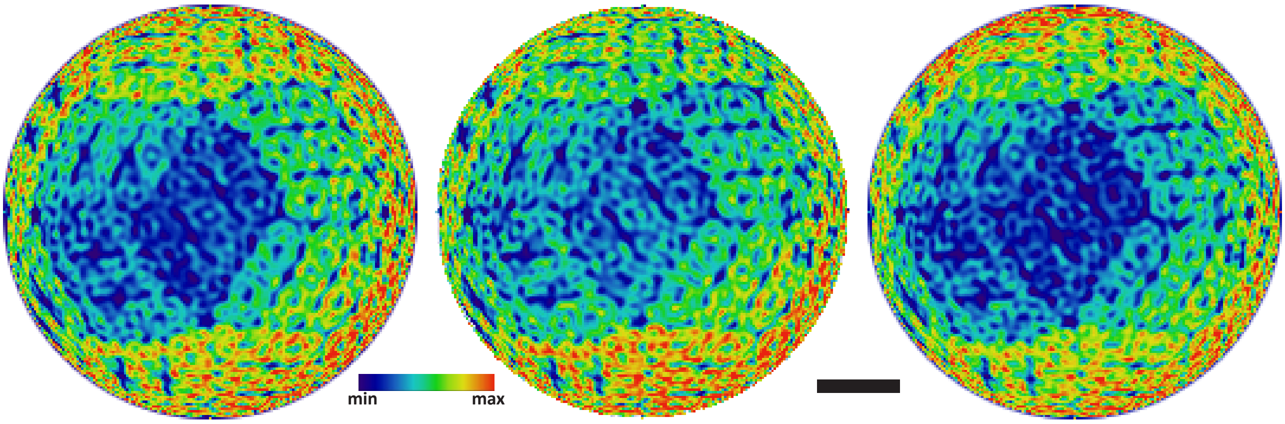

Fig. S17. Spherical shells of cryo-EM density values of asymmetric reconstructions of TBEV virions. The shells with the radius 222 nm are rainbow-colored based on the value of cryo-EM densities, ranging from blue (low) to red (high). Scale bar represents 10 nm. The shells of density are shown for the particles displayed in Fig. 2.

Table S1. Cryo-EM data collection, refinement, and validation statistics.

Data collection and processing	iTBEV spike SPA	iTBEV spike STA	iTBEV 2 pentamer missing	iTBEV 2 pent. missing 2	iTBEV altered 5-fold	TBEV asymmetric virion
Detector	Gatan K3	Gatan K3	Gatan K3	Gatan K3	Gatan K3	Gatan K3
Detector acquisition mode	counting	counting	counting	counting	counting	counting
Nominal Magnification	105,000	64,000	105,000	105,000	105,000	105,000
Voltage (kV)	300	300	300	300	300	300
Electron exposure on sample (e^-/Å^2)	44	148	44	44	44	48
Target defocus range (μm)	-3.5 – -1	-3.5 – -2	-3.5 – -1	-3.5 – -1	-3.5 – -1	-3.5 – -1
Calibrated pixel size (Å)	0.8336	1.346	0.8336	0.8336	0.8336	0.8336
Symmetry imposed	C1	C1	C1	C1	C1	C1
Number of collected movies	11,246	7913	11,246	11,246	11,246	13,122
Fractions per movie	40	4	40	40	40	40
Initial particle images	1,639,578	426,553	33,236	33,236	33,236	162,541
Final particle images	552,993	181,840	11,571	13,214	4499	73,881
Map resolution at $FSC_{0.143}$ (Å)	3.57	4.24	9.41	10.25	10.96	5.1
Map sharpening *B* factor (Å^2)	-115	-116	NA	NA	NA	-180
**Refinement and validation statistics**						
Model composition						
Non-hydrogen atoms (one protomer)	19,180					
Protein residues (one protomer)	2610					
Waters (one protomer)	0					
B factors						
Protein (Å^2)	35.4					
Waters (Å^2)	NA					
R.M.S. deviations						
Bond lengths (Å)	0.005					
Bond angles (°)	0.568					
Validation						
Molprobity score*	1.75 (100th percentile)					
Clashscore*	7.76 (97rd percentile)					
EMringer score	2.04					
map to model $FSC_{0.5}$ (Å)	3.6					
Rotamers						
Favored (%)	97.96					
Poor (%)	0.1					
Ramachandran plot						
Favored (%)	95.29					
Allowed (%)	4.63					
Poor (%)	0.08					
Accession codes						
PDB	9SWK	NA	NA	NA	NA	NA
EMDB	EMD-55311	EMD-55312	EMD-55313	EMD-55314	EMD-55315	EMD-55316

* values according to Molprobity.
